# Light regulates the degradation of the regulatory protein VE-1 in the fungus *Neurospora crassa*

**DOI:** 10.1186/s12915-022-01351-x

**Published:** 2022-06-27

**Authors:** María del Mar Gil-Sánchez, Sara Cea-Sánchez, Eva M. Luque, David Cánovas, Luis M. Corrochano

**Affiliations:** Departamento de Genética, Universidad de Sevilla, Reina Mercedes s/n, 41012 Seville, Spain

**Keywords:** Velvet complex, Light regulation, Protein degradation, COP9 signalosome

## Abstract

**Background:**

Fungi use light as an environmental signal to regulate developmental transitions that are key aspects of their biological cycles and that are also relevant for their dispersal and infectivity as plant or animal pathogens. In addition, light regulates the accumulation of photoprotective pigments, like carotenoids, and other secondary metabolites. Most fungal light responses occur after changes in gene transcription and we describe here a novel effect of light in the regulation of degradation of VE-1, a key component of the velvet complex, in the model fungus *Neurospora crassa*. The velvet complex is a fungal-specific protein complex that coordinates fungal development, secondary metabolism, and light regulation by interacting with other regulators and photoreceptors and modifying gene expression.

**Results:**

We have characterized the role of VE-1 during conidiation in *N. crassa*. In vegetative mycelia, VE-1 is localized in the cytoplasm and nuclei and is required for light-dependent transcription but does not interact with the photoreceptor and transcription factor WC-1. VE-1 is more stable in light than in darkness during asexual development (conidiation). We have shown that this light effect requires the blue-light photoreceptor WC-1. We have characterized the role of the proteasome, the COP9 signalosome (CSN), and the adaptor component of cullin-RING ubiquitin ligases, FWD-1, in the degradation of VE-1.

**Conclusions:**

We propose that this new effect of light allows the fungal cell to adapt quickly to changes in light exposure by promoting the accumulation of VE-1 for the regulation of genes that participate in the biosynthesis of photoprotective pigments.

**Supplementary Information:**

The online version contains supplementary material available at 10.1186/s12915-022-01351-x.

## Background

Developmental transitions in fungi are key to their success as plant and human pathogens. The development of appresoria by *Magnaporthe oryzae* and related fungi facilitates invasion of plant cells [1]. The production of conidia, spores, or ascospores facilitates dispersal and the colonization of new hosts by *Aspergillus fumigatus* and other pathogenic ascomycetes [2]. The development of resistance structures, sclerotia, facilitates later invasion of plants by *Botrytis cinerea* when environmental conditions are more appropriate [3]. Fungal development is regulated by environmental and internal signals. Asexual reproduction in *Neurospora crassa*, conidiation, is controlled by a circadian clock resulting in bands of conidia appearing approximately every 24 h [4]. Exposure to air, desiccation, lack of nutrients, and light regulate asexual and sexual development in many fungi [5–8]. These environmental signals are perceived and signaled into the cell by using signal transduction pathways that often include heterotrimeric G-proteins and their receptors [9–11], and photoreceptors [12, 13]. In *N. crassa*, light is perceived through a blue-light photoreceptor complex, the White Collar Complex (WCC), a photoreceptor, and transcription factor complex composed of the photoreceptor and Zn-finger protein WC-1 and the Zn-finger protein WC-2. The WCC transiently binds to promoters to activate transcription [14, 15]. WC proteins have been described in most fungi together with other photoreceptors like phytochromes, cryptochromes, and rhodopsins [12, 13]. In *Aspergillus nidulans*, WC proteins interact with the phytochrome in a light sensing complex capable of sensing red light (phytochrome) and blue light (WC proteins) [16, 17].

The regulation of protein turnover plays a key role in fungal development. Early studies uncovered the role of the COP9 signalosome (CSN) in fruiting body development during the sexual cycle of *A. nidulans* [18, 19]. Mutations in the genes encoding CSN subunits in *N. crassa* result in growth defects, alterations in conidation, and misregulation of the circadian clock [20]. The CSN is a deneddylase that removes the ubiquitin-like protein Nedd8 from modified proteins and is composed of eight subunits in *A. nidulans* and seven subunits in *N. crassa*, including the CSN-5/CsnE subunit responsible for the Nedd8 isopeptidase activity [21]. The deneddylase activity of the CSN is a key element in the regulation of the ubiquitin pathway of protein degradation by the proteasome. Proteins are targeted for degradation after labeling with ubiquitin units by cullin-RING ubiquitin ligases (CRLs) in the ubiquitin–proteasome pathway [22, 23]. In *N. crassa*, the degradation of FRQ, the key regulator of the circadian clock, is mediated by the F-box protein FWD-1, the adaptor component of a CRL that interacts with target proteins to promote their ubiquitylation and degradation by the proteasome [24]. FWD-1, together with SKP-1 and CUL-1, form a type of CRL named SCF (SKP Cullin F-box) that mediates FRQ ubiquitylation and degradation [24, 25]. The activity of CRLs, including SCF^FWD−1^, is regulated by removal of the ubiquitin-like protein Nedd8 by the CSN. Active CRLs require the deneddylation activity of the CSN since it promotes the recycling of CRL components for interaction with additional target proteins. In addition, deneddylation of CRLs by the CSN prevents the degradation of the CRLs by excessive autoubiquitylation [26–29]. Mutations that render the CSN inactive result in reduced amounts of FWD-1, and increased protein stability, as observed by the increased stability of FRQ in *N. crassa* strains with mutations in genes encoding CSN subunits [20, 25].

Fungi can produce many secondary metabolites, among them penicillin and other antibiotics, as well as enzymes and toxic chemicals such as the carcinogenic aflatoxin that help them thrive and facilitate their pathogenic lifestyles [30]. Other secondary metabolites, carotenoids and melanins, protect fungi from oxidative stress and UV radiation [31, 32]. Fungal development and secondary metabolism are often coordinated by environmental signals such as light [33]. A key element in the coordination of fungal development and secondary metabolism is the velvet complex [33, 34]. This fungal-specific protein complex was first identified in *A. nidulans* and is composed of the velvet proteins VeA and VelB, and the methyltransferase LaeA [35]. The velvet proteins VE-1 and VE-2, and a LaeA homolog, LAE-1, have been identified and characterized in *N. crassa* [36, 37]. Velvet proteins are defined by the velvet domain, a 150-amino acid domain involved in dimer formation with a structure that resembles the DNA-binding fold of the mammalian transcription factor NF-κB [38]. The velvet complex is proposed to regulate transcription by DNA binding to promoter regions and chromatin modification [39].

The activity of the velvet complex is regulated by the subcellular localization of its components, and by interactions with other regulatory proteins. In *A. nidulans*, VeA enters the nucleus as a heterodimer with VelB and interacts in the nucleus with LaeA to form the trimeric velvet complex [35]. Entry of VeA/VelB to the nucleus requires the importin KapA and is regulated by light. VeA is located in the nuclei when the fungus is grown in the dark, but light promotes the accumulation of VeA in the cytoplasm [40]. VeA interacts with kinases and methyltransferases that regulate its phosphorylation and the subcellular distribution of the protein [39, 41]. In addition, VeA interacts with the red-light photoreceptor phytochrome (FphA), presumably to coordinate fungal development and light regulation [16]. VeA, thus, is a molecular scaffold in several protein complexes that allows the integration of environmental signals to modulate gene transcription and development [42]. Protein complexes composed of velvet proteins regulate gene expression in other fungi. In the human pathogen *Histoplasma capsulatum*, two velvet proteins interact with the transcription factor Ryp1 to regulate the transcription of genes related to virulence and cell morphology [43].

In *N. crassa*, the VeA homolog, VE-1, interacts with VE-2 and LAE-1 to form a velvet complex in vegetative hyphae where it regulates secondary metabolism, and the accumulation of carotenoid pigments. In addition, the *N. crassa* velvet complex participates in the regulation of sexual development and the formation of aerial hyphae, the initial stage of asexual development (conidiation) [36]. Despite the regulatory role of the velvet complex in the coordination of environmental sensing, secondary metabolism, and development, there is little information regarding the presence and subcellular localization of the velvet complex during fungal development and how it regulates transcription.

Here, we characterize the role of VE-1 in the transcriptional control of secondary metabolism using carotenoid biosynthesis as a model, and we show that its action is independent of the blue-light photoreceptor and transcription factor WC-1. In addition, we show that a novel light-controlled degradation process regulates the presence of VE-1 in aerial hyphae during conidiation in *N. crassa*. The regulation by light of VE-1 degradation is a new mechanism that modulates the amount of VE-1 and its availability to form the regulatory velvet complex. Our results underscore the coordination between light and developmental stage in regulating the availability of VE-1 for the formation of the velvet complex to regulate asexual development in *N. crassa*.

## Results

### VE-1 is required for the accumulation of carotenoids and light-dependent transcription

**Fig. 1:**
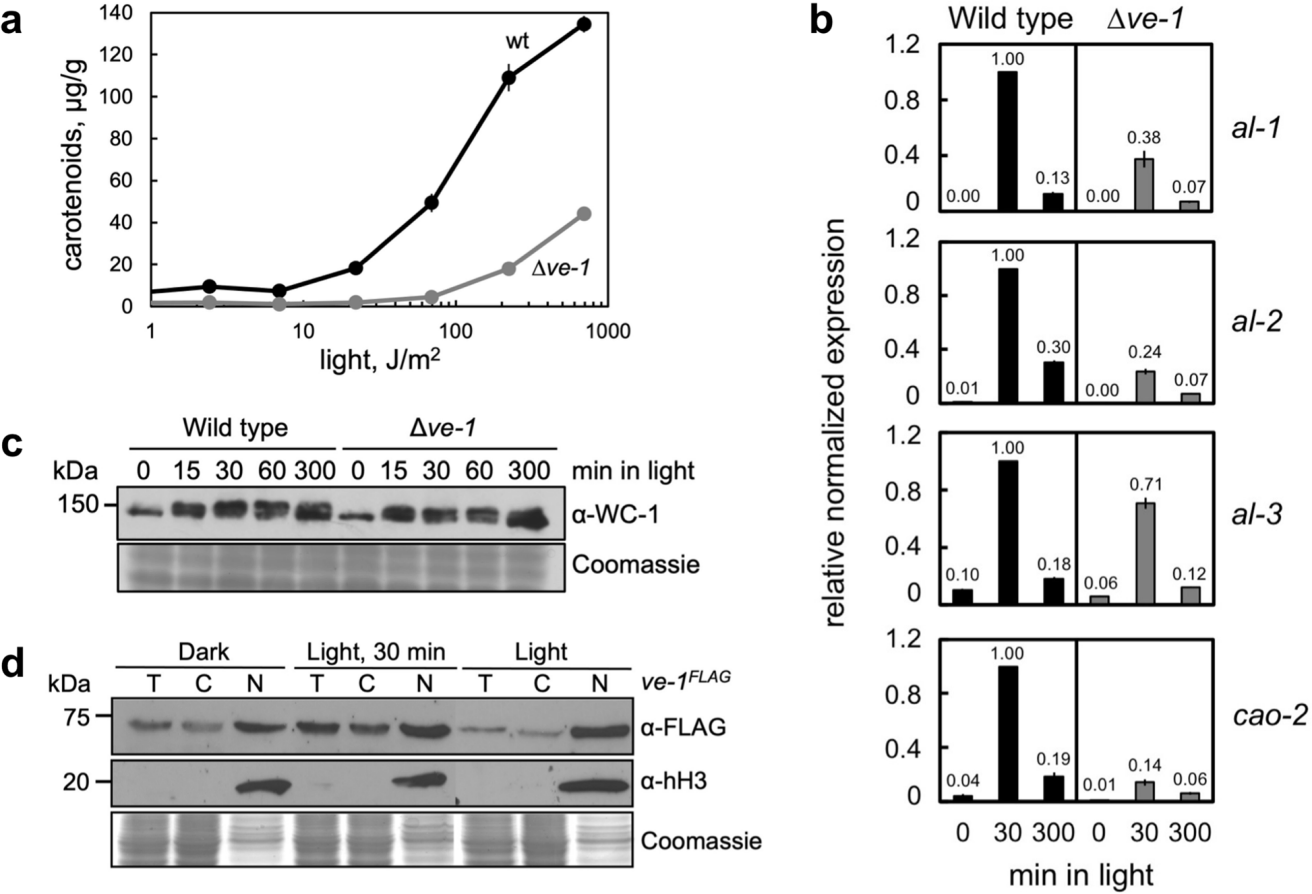
VE-1 regulates light-dependent transcription and the accumulation of carotenoids. **a** Effect of light intensity on the accumulation of carotenoids. Mycelia of the wild-type and Δ*ve-1* mutant strains were grown for 2 days in the dark at 22 °C and illuminated for 2 min prior to incubation for 24 h in the dark at 8 °C. The plot shows the average and standard error for six measurements for each light intensity. **b** Light-dependent transcription and photoadaptation. Wild-type and Δ*ve-1* mutant strains were grown for 2 days at 22 °C in the dark and then exposed to light during the times indicated prior to RNA purification and quantification by RT-PCR. The plots show the average and standard error of the mean of the relative mRNA accumulation in three independent experiments. The results from each PCR for each gene were normalized to the corresponding PCR for *tub-2* to correct for sampling errors. Then, the results were normalized to those obtained with the wild type after exposure to 30 min of light. **c** Light-dependent phosphorylation of the photoreceptor WC-1. Mycelial samples of the wild-type and the Δ*ve-1* mutant strains were grown for 2 days at 22 °C in the dark and then exposed to light during the times indicated. Total protein extracts were separated by SDS-PAGE, and hybridized with an antibody specific for WC-1. Two hundred micrograms of proteins was loaded per lane. Additional bands are due to the transient light-dependent WC-1 phosphorylation. As loading control, we used a Coomassie staining of each protein sample. **d** Subcellular localization of VE-1. Mycelial samples of the *ve-1*^*FLAG*^ strain were grown for 2 days at 30 °C in the dark, light, or grown in the dark and exposed to light during 30 min. Total protein samples (T), or samples enriched in cytoplasmic (C) or nuclear (N) proteins were separated by SDS-PAGE, and hybridized with antibodies specific for FLAG or histone H3. Seventy micrograms of proteins was loaded per lane. As loading control, we used a Coomassie staining of each protein sample

The velvet complex regulates the accumulation of the orange carotenoid neurosporaxanthin in vegetative mycelia of *N. crassa* as shown by the reduced accumulation of carotenoids after exposure to light [36, 37]. The decrease in carotenoid accumulation was reverted by transformation with a wild-type copy of *ve-1* (Additional file 1: Fig. S1a, b). In addition, VE-1 regulated the accumulation of carotenoids in conidia (Additional file 1: Fig. S2a, b). Light induces the accumulation of carotenoids in mycelia after the activation by the WCC of the genes encoding the biosynthetic enzymes (*al-1*, *al-2*, *al-3*, and *cao-2*) [44]. The role of the velvet complex in the regulation of carotenoid biosynthesis and the possible interaction with the WCC are not understood. VE-1 regulates the expression of 15–20% of all *N. crassa* genes in vegetative mycelia, and the *ve-1* mutant had a small reduction in the accumulation of the mRNAs for *al-2* (encoding the phytoene synthase) and *cao-1* (encoding a carotenoid oxygenase) under standard growth conditions [36]. The absence of VE-1 led to a tenfold reduction in the capacity to perceive light by vegetative hyphae as the threshold for light-dependent accumulation of carotenoids increased to 100 J/m^2^ in the Δ*ve-1* mutant compared to the wild-type threshold of 10 J/m^2^ (Fig. 1a).

To figure out whether the reduction in the capacity to perceive light in the Δ*ve-1* mutant was caused by alterations in the amount or activity of the photoreceptor WC-1 expression levels of light-dependent genes were analyzed. Light activates transiently the genes for the biosynthetic enzymes, photoadaptation, and the light-dependent mRNA accumulation of these genes was reduced in the absence of VE-1 (Fig. 1b). A reduction in light-dependent mRNA accumulation has been observed for other light-regulated genes in the Δ*ve-1* mutant, indicating that this effect on transcription is not limited to carotenoid genes (Additional file 1: Fig. S2c). Photoadaptation correlates with a transient light-dependent phosphorylation of WC-1 [45]. However, the loss of sensitivity to light and the reduced light-dependent transcription of carotenoid genes were not due to changes in the amount or light-dependent phosphorylation of the photoreceptor WC-1 (Fig. 1c). Our results suggested, instead, a direct regulatory role of VE-1, and the velvet complex, on light-dependent transcription in *N. crassa* nuclei. In order to confirm the nuclear localization of VE-1, we created a strain with VE-1 tagged with the FLAG epitope for the detection of the protein using a FLAG-specific antibody (Additional file 1: Fig. S2a). The new allele (*ve-1*^*FLAG*^) was under the control of the *ve-1* native promoter as it replaced the original gene and will be used as the wild-type strain in the rest of this work. The *ve-1* gene was weakly activated by light in vegetative hyphae (Additional file 1: Fig. S2d), and the tagged VE-1 protein was detected in similar amounts in mycelia kept in the dark or exposed to light in the wild-type strain or in strains with mutations in the genes for the blue-light photoreceptors WC-1 or VVD (Additional file 1: Fig. S2e). We detected VE-1 in the cytoplasmic and nuclear fractions, and the subcellular distribution of VE-1 did not change with light in the wild-type strain (Fig. 1d), or in strains with mutations in the genes for the blue-light photoreceptors (Additional file 1: Fig. S2f). These results supported the proposal that light-dependent activation of transcription in *N. crassa* requires the WCC and the velvet complex.

### Light prevents the degradation of VE-1 in aerial hyphae and allows the accumulation of VE-1 in the nucleus

**Fig. 2:**
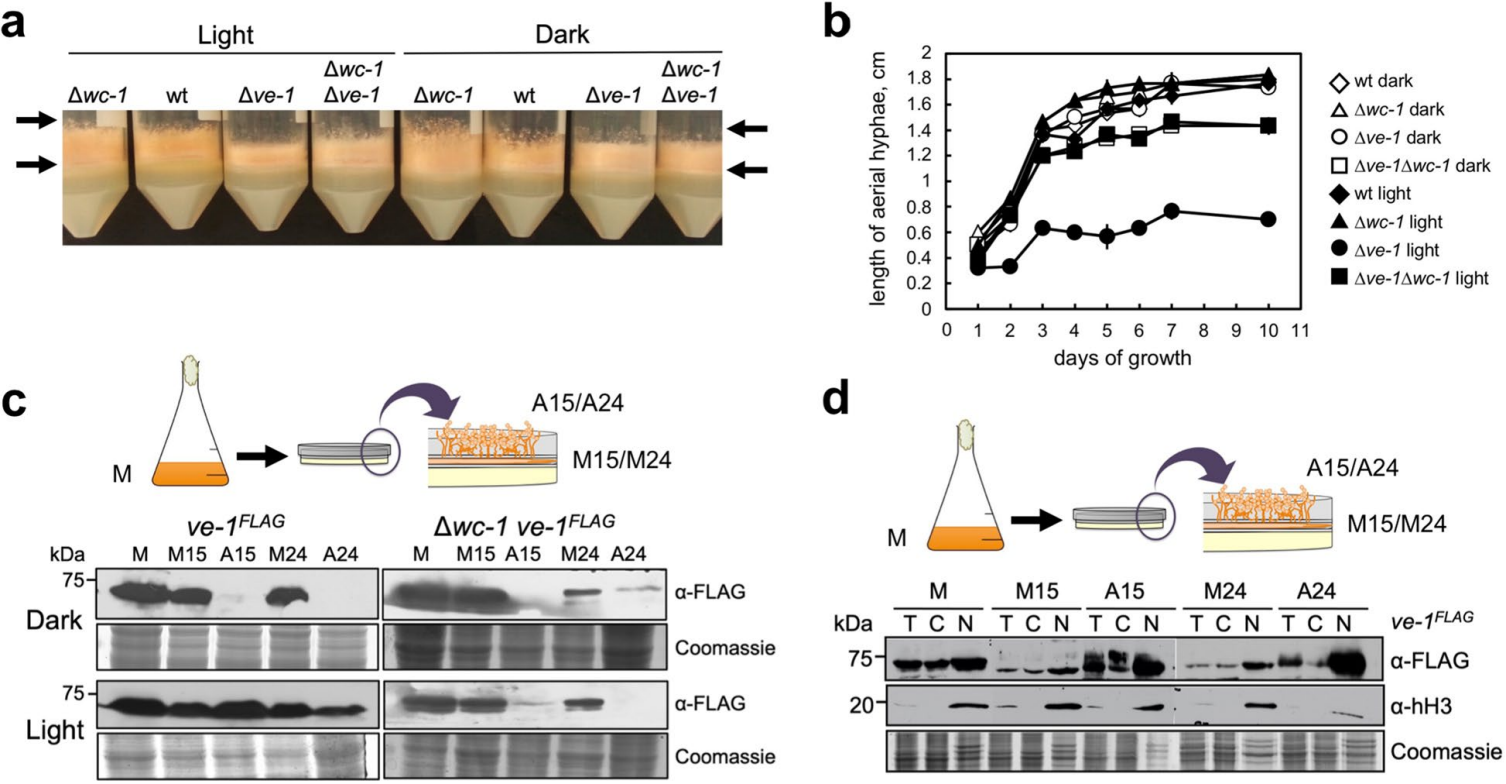
Light regulates the accumulation of VE-1 during conidiation. **a** VE-1 regulates the growth of aerial hyphae. Aerial hyphae of the wild-type, Δ*ve-1*, Δ*wc-1*, and Δ*wc-1* Δ*ve-1* strains after 3 days of growth in minimal agar at 30 °C in dark or light. The length of aerial hyphae growing up from vegetative mycelia in two representative cultures is indicated by arrows. **b** Length of aerial hyphae in each strain after growth at 30 °C in dark or light. Average and standard error of the mean in 3–5 independent experiments. **c** Accumulation of VE-1 during conidiation. Upper part. Protein samples from cultures kept in the dark or light were obtained from mycelia growing vegetatively in liquid media (M), or as supporting vegetative mycelia after 15 h (M15) or 24 h (M24) of transfer from liquid media to the surface of an agar plate with minimal media to induce conidiation. Conidiating aerial hyphae were collected after 15 h (A15) or 24 h (A24) of the induction of conidiation. We used the *ve-1*^*FLAG*^ and the Δ*wc-1 ve-1*^*FLAG*^ strains. Bottom part. Proteins were separated by SDS-PAGE, and hybridized with an antibody specific for FLAG. One hundred micrograms of proteins was loaded per lane. As loading control, we used a Coomassie staining of each protein sample. **d** VE-1 accumulates in the nuclear fraction during conidiation. Upper part. We collected samples of the *ve-1*^*FLAG*^ strain grown in the light from vegetative mycelia (M), supporting vegetative mycelia (M15) or (M24), and aerial hyphae (A15) or (A24). Bottom part. Total protein samples (T), or samples enriched in cytoplasmic (C) or nuclear (N) proteins were separated by SDS-PAGE, and hybridized with antibodies specific for FLAG or histone H3. Seventy micrograms of proteins was loaded per lane. As loading control, we used a Coomassie staining of each protein sample

VE-1 and the velvet complex participate in the regulation of asexual development [36]. The absence of *ve-1* led to short aerial hyphae, an early stage of conidiation [46], and this defect was reverted by transformation with the wild-type *ve-1* gene (Additional file 1: Fig. S1a, c). The reduction in the length of aerial hyphae in the Δ*ve-1* mutant was observed only in cultures kept in light. The effect of light on aerial hyphae length required the photoreceptor WC-1, since we did not detect any difference in the length of aerial hyphae of the Δ*ve-1* Δ*wc-1* strain kept in dark or light (Fig. 2a,b). The absence of *ve-1* led to a reduction in the accumulation of conidia compared to the wild type, in particular when the Δ*ve-1* strain was grown in light (Additional file 1: Fig. S3), suggesting that the absence of VE-1 led to a light-dependent inhibition of conidiation by inhibiting the growth of aerial hyphae. We hypothesized that VE-1 should play its regulatory role in the nuclei of aerial hyphae and we used the strain with the *ve-1*^*FLAG*^ allele to monitor the abundance and subcellular localization of VE-1 during conidiation. For these experiments, we first incubated conidia from each strain in liquid media to promote vegetative growth for 24 h. We then transferred the hyphae from liquid media to the air-exposed surface of a petri dish with solid minimal agar to induce conidiation and covered the vegetative mycelia with filter paper. After incubation in dark or light, we collected the aerial hyphae that grew through the filter paper and the supportive vegetative mycelia under the filter paper for RNA or protein extraction, and characterization [47] (Additional file 1: Fig. S4a).

We detected VE-1 in vegetative mycelia kept in dark or light, but we observed that light played a key role on the accumulation of VE-1 during conidiation. We could not detect VE-1 in aerial hyphae that had grown in the dark, but VE-1 was clearly observed in samples of aerial hyphae that had been exposed to light (Fig. 2c). The absence of VE-1 in aerial hyphae kept in the dark was surprising since we detected similar amounts of *ve-1* mRNA in mycelia and aerial hyphae in cultures that had been kept in dark or light (Additional file 1: Fig. S4b). The presence of a PEST domain (a protein segment rich in proline, glutamic acid, serine, and threonine) in VE-1 [37] suggested that VE-1 could be subjected to degradation by the proteasome. We, thus, hypothesized that VE-1 could be degraded in aerial hyphae kept in the dark and that light perceived through the photoreceptor WC-1 could prevent the degradation of VE-1 in aerial hyphae. In order to confirm this hypothesis, we performed similar experiments in a Δ*wc-1* strain. This strain accumulated very little VE-1 in aerial hyphae in all conditions, confirming the key role of light in preventing VE-1 degradation in aerial hyphae (Fig. 2c). VE-1 was detected in both cytoplasmic and nuclear fractions, but the amount of VE-1 in the nuclear fraction of light-exposed aerial hyphae increased when compared to mycelial samples (Fig. 2d).

Our results suggested that VE-1 was degraded in aerial hyphae in the dark and that the degradation of VE-1 was reduced by exposure to light perceived through WC-1. We hypothesized that a light-regulated degradation process controls the abundance of VE-1 in aerial hyphae of *N. crassa* allowing the accumulation of VE-1 in nuclei to regulate transcription during development.

### VE-1 is degraded by the proteasome and light regulates the degradation

**Fig. 3:**
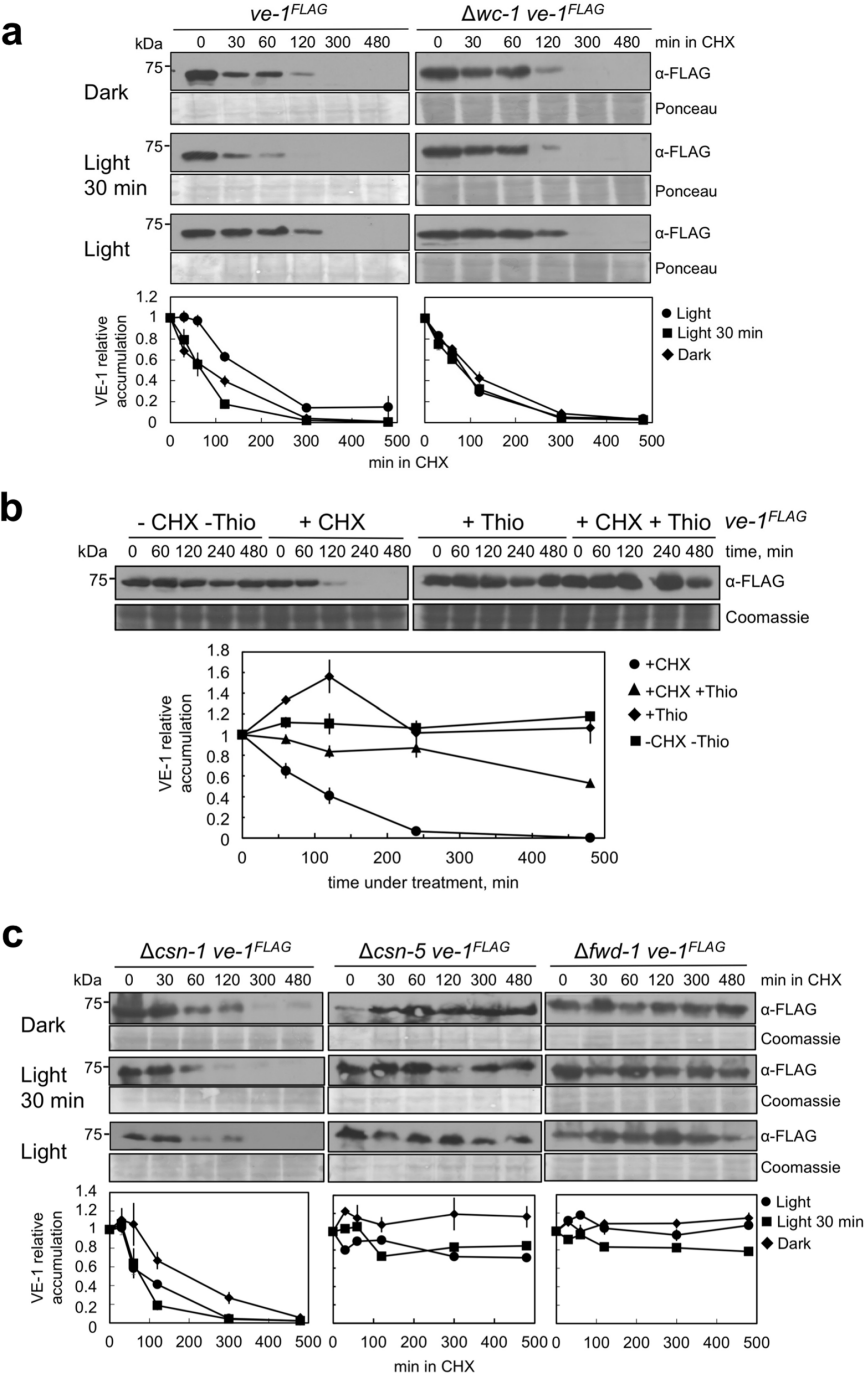
The degradation of VE-1 by the proteasome is regulated by light. **a** Regulation by light of VE-1 degradation. Cultures of vegetative mycelia of the *ve-1*^*FLAG*^ and the Δ*wc-1 ve-1*^*FLAG*^ strains were grown at 30 °C in liquid media for 24 h in the dark, light or exposed to 30 min of light, then cycloheximide was added to the cultures, and samples were collected at different times. Proteins were separated by SDS-PAGE, and hybridized with an antibody specific for FLAG. Seventy micrograms of proteins was loaded per lane. As loading control, we used a Ponceau staining of each protein sample. Each hybridization was quantified using as reference the amount of VE-1 detected at time point 0 (before addition of cycloheximide). The plot shows the average and standard error of four independent experiments. **b** The proteasome inhibitor thiolutin prevents the degradation of VE-1. Cultures of vegetative mycelia of the *ve-1*^*FLAG*^ strain were grown at 30 °C in liquid media and exposed to 30 min of light, then cycloheximide and/or thiolutin were added to the cultures, and samples were removed at different times. Cultures without any additional chemical added were used as controls. Proteins were separated by SDS-PAGE, and hybridized with an antibody specific for FLAG. Seventy micrograms of proteins was loaded per lane. As loading control, we used a Coomassie staining of each protein sample. The hybridization was quantified using as reference the amount of VE-1 detected at time point 0 (before addition of cycloheximide, thiolutin or both). The plot shows the average and standard error of three independent experiments. **c** FWD-1 and the CSN participate in the degradation of VE-1. Cultures of vegetative mycelia of the Δ*csn-1 ve-1*^*FLAG*^, Δ*csn-5 ve-1*^*FLAG*^, and Δ*fwd-1 ve-1*^*FLAG*^ strains were grown at 30 °C in liquid media for 24 h in the dark, light, or exposed to 30 min of light, then cycloheximide was added to the cultures, and samples were removed at different times. Proteins were separated by SDS-PAGE and hybridized with an antibody specific for FLAG. Seventy micrograms of proteins was loaded per lane. As loading control, we used a Coomassie staining of each protein sample. Each hybridization was quantified using as reference the amount of VE-1 detected at time point 0 (before addition of cycloheximide). The plot shows the average and standard error of 2–3 independent experiments

The mechanism of light-regulated degradation of VE-1 was assayed by treating *N. crassa* vegetative mycelia with cycloheximide, an inhibitor of protein synthesis. We could not detect VE-1 after 2 h in cycloheximide unlike a stable protein like the nucleolar protein homologous to yeast Nop1p (Additional file 1: Fig. S5a). The half-life of VE-1 in cycloheximide was about 100 min, but we observed that the degradation of VE-1 could be modulated by light resulting in different degradation kinetics (Fig. 3a). Continuous exposure to light reduced the degradation of VE-1 as observed in particular after 120 min in cycloheximide, while the degradation of VE-1 increased after 30 min of light when compared to cultures kept in the dark (Fig. 3a). The effect of light modulating VE-1 stability required the photoreceptor WC-1 since the kinetic of VE-1 degradation in the Δ*wc-1* strain did not change under any light regime (Fig. 3a). WC-1 is a very stable protein (Additional file 1: Fig. S5b), and it does not interact with VE-1 in co-immunoprecipitation assays (Additional file 1: Fig. S5c) likely ruling out any direct regulation of WC-1 on VE-1 stability. In order to characterize the degradation of the other components of the velvet complex, VE-2 and LAE-1, we used *N. crassa* strains carrying *ve-2*^*HA*^ or *lae-1*^*FLAG*^ alleles for detection of VE-2 or LAE-1 with antibodies specific for HA or FLAG, respectively [36]. Unlike VE-1, we observed the accumulation of VE-2 and LAE-1 after long incubations in cycloheximide (480 min) suggesting that VE-1 was the only component of the velvet complex subjected to degradation when protein synthesis was blocked (Additional file 1: Fig. S5d). Our results confirmed that light regulates the amount of VE-1 by controlling its degradation in vegetative mycelia, but the role of the photoreceptor WC-1 remains to be understood. Identifying the mechanism of VE-1 degradation will help to understand the interactions between light, protein degradation, and VE-1 activity.

To confirm the role of the proteasome in the degradation of VE-1, we used thiolutin, an inhibitor of JAB1/MPN/Mov34 (JAMM) metalloproteases, such as Rpn11, the deubiquitinating enzyme of the 19S proteasome lid, and Csn5, the deneddylase of the COP9 signalosome (CSN) that regulates cullin-RING ubiquitin ligases. Thiolutin inhibits the ubiquitin–proteasome system (UPS) and protein turnover leading to increased protein stability [48]. Incubation of *N. crassa* vegetative mycelia with cycloheximide and thiolutin resulted in increased stability of VE-1 when compared with samples incubated with cycloheximide only (Fig. 3b). This result supported the proposal that VE-1 was degraded by the proteasome.

### The degradation of VE-1 requires the COP9 signalosome and the adaptor protein FWD-1

We then examined how VE-1 is targeted for degradation by the proteasome. The key role of the CRL adaptor protein FWD-1 on FRQ stability, and the circadian clock regulation on conidiation prompted us to characterize the role of FWD-1 and the CSN on the stability of VE-1. We created strains with deletions in *fwd-1*, *csn-1*, and *csn-5* that, in addition, carried the *ve-1*^*FLAG*^ allele for detection of VE-1 in protein extracts by crossing mutant strains with the wild-type strain carrying the *ve-1*^*FLAG*^ allele, and characterizing the progenies to identify strains with mutant and *ve-1*^*FLAG*^ alleles. We selected strains with mutations in *csn-5* and *csn-1* as two representatives of the main subunits of the CSN. CSN-5 is the JAMM-domain subunit of the CSN responsible for Nedd8 cleavage from cullin proteins, and CSN-1 is one of the several Proteasome, COP9 signalosome, and eukaryotic initiation factor 3 (PCI)-domain proteins in the CSN [20, 49]. Vegetative mycelia from the three mutant strains were incubated with cycloheximide and the stability of VE-1 was assayed in protein extracts. VE-1 was degraded after incubation with cycloheximide in the Δ*csn-1* strain following a kinetics and light regulation similar to that observed previously in the wild-type strain, but mutations in *csn-5* or *fwd-1* prevented the degradation of VE-1, even after incubations of 8 h in cycloheximide (Fig. 3c). It seems that the absence of CSN-1 did not impair the capacity of the CSN to regulate the degradation of VE-1, but the Nedd8 cleavage activity provided by CSN-5 was a key element for the role of the CSN in the regulation of VE-1 degradation. These mutations did not modify the light-dependent phosphorylation of WC-1 or the subcellular distribution of VE-1 in vegetative mycelia (Additional file 1: Fig. S6), only the stability of VE-1. Our results suggest that the degradation of VE-1 requires the SCF^FWD−1^ and its adequate regulation by the CSN.

### VE-1 accumulates in aerial hyphae and conidia of strains with mutations in genes of the COP9 signalosome or the adaptor protein FWD-1

**Fig. 4:**
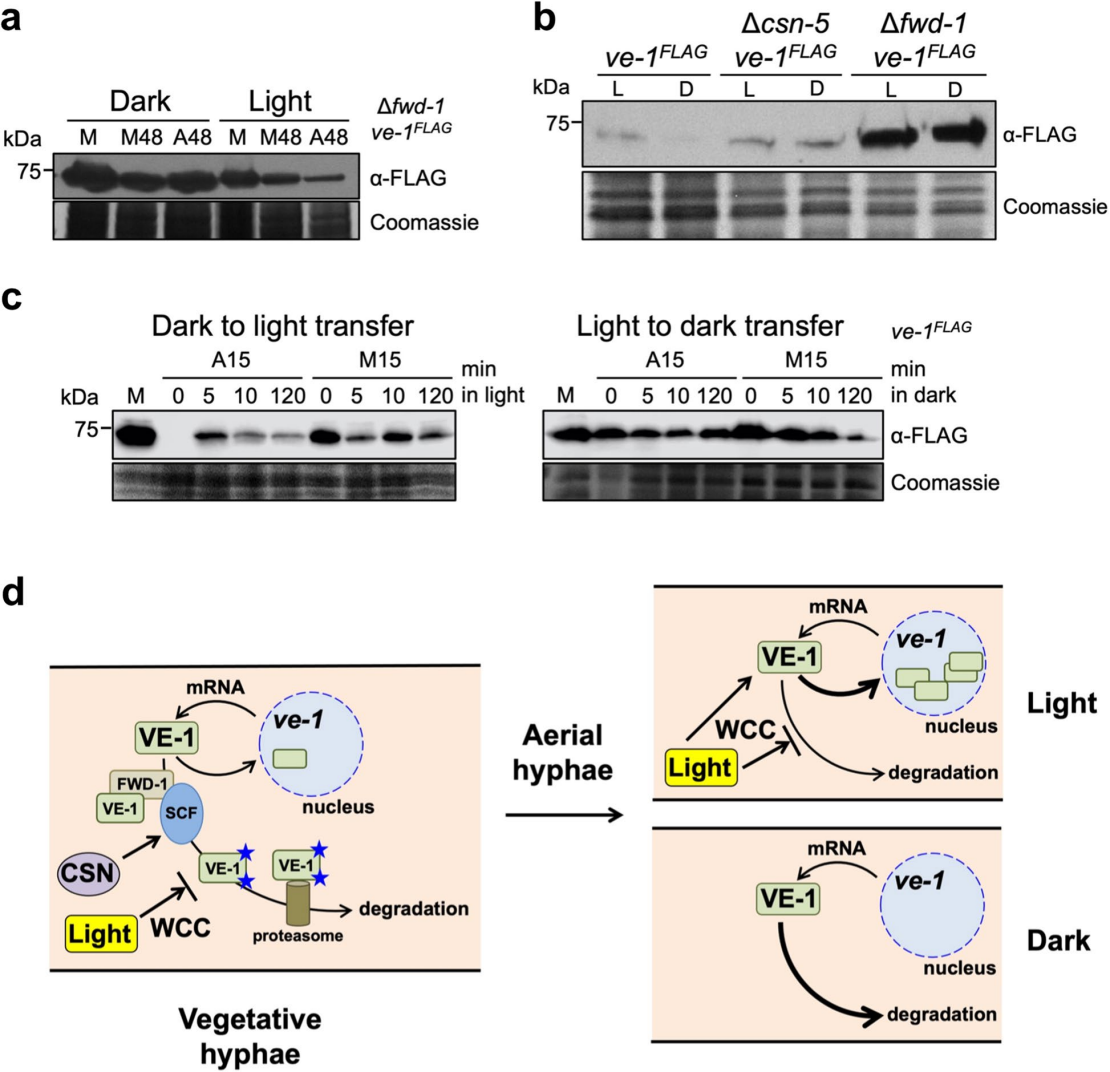
Light regulates the accumulation of VE-1 in aerial hyphae by preventing its degradation. **a** The degradation of VE-1 in aerial hyphae requires FWD-1. Protein samples from cultures of the Δ*fwd-1 ve-1*^*FLAG*^ strain kept in the dark or light were obtained from mycelia growing vegetatively in liquid media (M), or as supporting vegetative mycelia after 48 h (M48) of transfer from liquid media to the surface of an agar plate with minimal media to induce conidiation. Conidiating aerial hyphae were collected after 48 h (A48) of the induction of conidiation. Proteins were separated by SDS-PAGE and hybridized with antibodies specific for FLAG. One hundred micrograms of proteins was loaded per lane. As loading control, we used a Coomassie staining of each protein sample. **b** The degradation of VE-1 in conidia requires FWD-1 and an active CSN. Protein samples were isolated from conidia of the *ve-1*^*FLAG*^, Δ*csn-5 ve-1*^*FLAG*^, and Δ*fwd-1 ve-1*^*FLAG*^ strains isolated from cultures kept in the dark or light. Proteins were separated by SDS-PAGE and hybridized with antibodies specific for FLAG. Fifty micrograms of proteins was loaded per lane. As loading control, we used a Coomassie staining of each protein sample. **c** Light promotes the accumulation of VE-1 during conidiation. Protein samples from cultures of the *ve-1*^*FLAG*^ strain kept in the dark or light were obtained from mycelia growing vegetatively in liquid media (M), or as supporting vegetative mycelia after 15 h (M15) of transfer from liquid media to the surface of an agar plate with minimal media to induce conidiation. Conidiating aerial hyphae were collected after 15 h (A15) of the induction of conidiation. For the dark to light transfer or light to dark transfer, the M15 or A15 samples were incubated in light or dark, respectively, for the indicated times after 15 h of induction of conidiation in dark or light, respectively, and collected for protein purification. Proteins were separated by SDS-PAGE and hybridized with an antibody specific for FLAG. One hundred micrograms of proteins was loaded per lane. As loading control, we used a Coomassie staining of each protein sample. **d** A model for the regulation of VE-1 degradation during conidiation and light. VE-1 accumulates in the cytoplasm and nuclei of vegetative hyphae following transcription and translation. VE-1 is degraded by the proteasome in a process that requires interaction with the SCF^FWD−1^ and ubiquitylation (symbolized by stars) and is regulated by the CSN and light. During the development of aerial hyphae VE-1 is highly degraded but light reduces VE-1 degradation and promotes its translocation to the nucleus for gene regulation

The experiments that assayed the stability and degradation of VE-1 in the wild-type strain and mutants were carried out in vegetative mycelia incubated in liquid media with cycloheximide. Since we did not detect VE-1 in aerial hyphae kept in the dark, we hypothesized that SCF^FWD−1^ and the CSN had a key role in the degradation of VE-1 in aerial hyphae and that this degradation would be reduced by exposure to light. Thus, we expected an increased accumulation of VE-1 in aerial hyphae of strains with mutated *csn* subunit genes or *fwd-1*. Mutants in subunits of the CSN or in *fwd-1* show slow growth and delayed conidiation [20, 24, 25], presumably due to the major alteration in protein stability caused by these mutations. We grew the Δ*fwd-1* mutant mycelia for 48 h to induce conidiation and to isolate sufficient aerial hyphae for protein extraction and detection, but its slow growth rate prevented direct comparisons with the wild-type strain. However, we could detect VE-1 in aerial hyphae of the Δ*fwd-1* mutant strain that had been incubated in the dark or light, unlike our previous observations with aerial hyphae of the wild-type strain (Figs. 4a and 2c). In addition, we detected similar amounts of VE-1 in conidia isolated from dark- or light-grown Δ*fwd-1* or Δ*csn-5* mutant strains (Fig. 4b). As expected, the amount of VE-1 in conidia from the wild-type strain was higher in samples isolated from cultures kept in the light, than in the dark (Fig. 4c). Our results supported the proposal that the reduction in the amount of VE-1 in aerial hyphae kept in the dark was due to its degradation by the proteasome in a process regulated by SCF^FWD−1^ and the CSN.

### The reduction of VE-1 degradation by light allows rapid accumulation of VE-1 after dark to light transitions

Our results show that VE-1 protein turnover is regulated by light. Therefore, the biological role of this light control was investigated. Light often activates fungal transcription producing the accumulation of light-dependent mRNAs and proteins [12, 13]. However, the accumulation of light-induced proteins, and their biological effect, are likely to occur several minutes after the onset of illumination. In *N. crassa*, maximum mRNA accumulation from early light-responsive genes is observed with exposures of 15–30 min of light [44]. Short exposure times, for example 5 s, activate transcription of the conidiation genes *con-10* and *con-6*, but maximum mRNA accumulation was observed after 45 min of further incubation in the dark [50]. It is possible that light-dependent transcription and translation is not sufficient as a response to sudden changes in light exposure or intensities in natural habitats, such as the surface of burned trees where conidiating mycelia of *Neurospora* species are often observed [51, 52]. We can hypothesize that the regulation by light of VE-1 degradation could be a novel mechanism that ensures the presence of VE-1 for further transcriptional regulation during the development of conidia after sudden exposures to light. We thus would expect quick changes in the accumulation of VE-1 in aerial hyphae after a transition from dark to light. In order to prove this hypothesis, we incubated aerial hyphae of the wild-type strain for 15 h in the dark and then transferred the cultures to light for a variable time, from 5 to 120 min, prior to sample isolation and protein extraction. As expected, VE-1 was detected in vegetative mycelial samples but not in aerial hyphae after 15 h of growth in the dark. However, 5 min of further incubation in light was sufficient to prevent degradation of VE-1, allowing the detection of the protein in aerial hyphae (Fig. 4c). The opposite experiment, the transfer of light-exposed aerial hyphae to the dark, always led to accumulation of VE-1, at least up to 120 min of incubation in the dark (Fig. 4c). Our results suggest that the light-dependent regulation of VE-1 degradation is a novel mechanism that ensures the presence of VE-1 to regulate transcription during the response to light in the aerial hyphae of *N. crassa*.

## Discussion

The velvet complex coordinates fungal development, secondary metabolism, and light responses by regulating gene expression (33, 34). In *N. crassa*, the three components of the velvet complex (VE-1, VE-2, and LAE-1) interact in vegetative mycelia to regulate transcription [36], and we show here that the accumulation of VE-1 during the early stage of asexual development requires light exposure.

The disruption of the velvet complex by deletion of *ve-1* led to changes in the sensitivity to light as observed by the light-dependent accumulation of carotenoids, and a reduction in the light-dependent accumulation of mRNAs for carotenoid genes. We did not observe any changes in the amount or light-dependent phosphorylation of the photoreceptor WC-1 in the Δ*ve-1* strain, ruling out a direct regulation of VE-1 on *wc-1* transcription or WC-1 regulation. Our observations suggest that the WCC and the velvet complex participate in light-dependent transcription in the *N. crassa* nuclei.

The development of conidia requires an active velvet complex regulating transcription during vegetative growth prior to the initial stages of conidiation, the growth of aerial hyphae away from the solid surface into open air. Our observation that VE-2 and LAE-1 are stable proteins in cycloheximide, unlike VE-1, suggests that VE-1 is the limiting component in the velvet complex. The reduction in the amount of VE-1 that we detected in aerial hyphae kept in the dark suggests a reduction in the amount of the velvet complex for regulatory purposes once conidiation has started. A reduction in the amount of VeA has been observed during both sexual and asexual development in *A. nidulans*, and the degradation of VeA was prevented by gene deletions of the deubiquitinase UspA or the F-box protein Fbx23 [53]. A possible role for the *N. crassa* homolog of UspA in the regulation of VE-1 ubiquitylation has not been demonstrated, but these observations suggest a similar mechanism requiring the degradation of VE-1/VeA during the development of two ascomycete fungi. Despite these similarities, there are differences in the regulation by light of the localization of *A. nidulans* VeA and *N. crassa* VE-1. VeA is localized in the nucleus in the dark but not in the light [40], and we observed VE-1 in the cytoplasmic and nuclear fractions in both conditions, dark and light, in vegetative mycelia. The other components of the velvet complex in *N. crassa*, VE-2 and LAE-1, are also observed in cytoplasmic and nuclear fractions in vegetative mycelia, and their subcellular localization is not regulated by light [36]. It seems that the regulatory effect of light on the *N. crassa* velvet complex operates on the stability of VE-1 only. The stability or subcellular localization or other fungal VeA homologs have not been characterized, but their conserved role as regulators of fungal development, secondary metabolism, and pathogenesis [33, 34] suggests that the basic features of the velvet complex in *N. crassa* or *A. nidulans* will be shared by other fungi.

Our observation that light prevents the degradation of VE-1 in aerial hyphae suggested a mechanism to ensure that VE-1 was available, together with the rest of components of the velvet complex, for a full response to light. We have shown that the other components of the velvet complex, VE-2 and LAE-1, accumulate in aerial hyphae in dark and light (Cea-Sánchez S, Corrochano-Luque M, Gutiérrez G, Glass NL, Cánovas D, Corrochano LM: Transcriptional regulation by the velvet protein VE-1 during asexual development in the fungus *Neurospora crassa*, submitted), further supporting the proposal that VE-1 is the limiting component of the velvet complex in aerial hyphae. We have shown that VE-1 is degraded by the proteasome in a process that requires the adaptor protein FWD-1 and is regulated by the CSN. Our results can be summarized in a model (Fig. 4d) that shows how *ve-1* mRNA is transcribed and translated in vegetative hyphae so that VE-1 accumulates in both cytoplasm and nuclei for transcriptional regulation. Cytoplasmic VE-1 is subjected to degradation by the proteasome in a process that requires SCF^FWD−1^ and is regulated by the CSN and light. In vegetative hyphae, continuous transcription and translation provides sufficient VE-1 to counteract the degradation by the proteasome so that a constant supply of VE-1 is always present for regulatory purposes. During the development of aerial hyphae degradation of VE-1 is more prevalent, and VE-1 is barely observed in aerial hyphae in the dark. The presence of light, however, is a signal that reduces the degradation of VE-1 so that the protein can be quickly translocated to the nucleus to interact with the other components of the velvet complex to regulate transcription.

Light could regulate the degradation of VE-1 by several mechanisms. We have shown that the photoreceptor WC-1 and VE-1 do not interact, at least in vegetative mycelia, but they could interact weakly, or through other protein partners, to regulate the degradation of VE-1 by light. In *A. nidulans*, VeA interacts with the WC-2 homolog, LreB, and the phytochrome [16] but pull down experiments with VE-1, VE-2, or LAE-1 did not identify any interaction with the WCC or other photoreceptors in *N. crassa* vegetative mycelia [36]. These results do not rule out the possibility of weak interactions among the velvet complex and photoreceptors in *N. crassa*.

The genes encoding the proteins that participate in the degradation of VE-1 (FWD-1, SKP-1, CUL-1, and the CSN) are not regulated by light or conidiation (Cea-Sánchez S, Corrochano-Luque M, Gutiérrez G, Glass NL, Cánovas D, Corrochano LM: Transcriptional regulation by the velvet protein VE-1 during asexual development in the fungus *Neurospora crassa*, submitted), ruling out a possible transcriptional control in this process. However, we can hypothesize that light could provide protective posttranslational modifications in VE-1 and/or interactions with proteins that would prevent its degradation by the proteasome. In *A. nidulans*, the phytochrome FphA participates in a signal transduction pathway that leads to the light-dependent phosphorylation of the mitogen-activated protein (MAP) kinase SakA and its shuttling into nuclei for regulatory purposes [54]. It is conceivable that similar light-dependent phosphorylations or other posttranslational modifications of VE-1 may impair its degradation by the proteasome.

The production of secondary metabolites in *N. crassa* is very limited, unlike in other ascomycetes [30], but the velvet complex suppresses the accumulation of the siderophore coprogen under iron starvation conditions [36]. We have shown that VE-1 is required for full accumulation of mRNAs from *albino* and other genes required for the biosynthesis of the protective carotenoid pigments after light exposure, and we propose that full protection from light would require the transcriptional activity of VE-1 as soon as possible after the onset of illumination. Light plays a key regulatory role in the biology of *N. crassa* and other filamentous fungi. We propose that the novel mechanism that we have uncovered allows *N. crassa* to quickly change the amount of VE-1 and, perhaps, other regulatory proteins for better adaptation to changing illumination in nature.

## Conclusions

We have shown that the degradation of VE-1 is regulated by light during asexual development, and we propose that this mechanism allows a quick response to light by promoting the accumulation of VE-1 for transcriptional regulation. We propose that the regulation by light of protein degradation is a novel mechanism that allows fungal cells to adapt to changes in light exposure and intensity in nature.

## Methods

### Strains and culture conditions

Fungal strains used in this work are listed in Additional file 2: Table S1. For plasmid manipulations, we used *E. coli* DH5a. For gene tagging in *N. crassa*, we used the *S. cerevisiae* strain FY834 (MATα *his3Δ200 ura3-52 leu2Δ1 lys2Δ202 trp1Δ63*). We followed standard procedures and protocols for *N. crassa* strain manipulation and growth media preparation [55]. See also, the *Neurospora* protocol guide (http://www.fgsc.net/Neurospora/NeurosporaProtocolGuide.htm).

For the light induction experiments, 10^6^ conidia were inoculated on 25 ml Vogel’s liquid medium in 90-mm plates, which were incubated in complete darkness at 22 °C for 48 h. The cultures were then exposed to light for the times indicated. For carotenoid induction, plates were exposed to light for 2 min, and then incubated at 8 °C for 24 h prior to collection. White light exposure with different intensities was obtained with a quartz halogen lamp installed in a slide projector passed through a filter holder with two heat filters and neutral-density filters, as required to obtain the desired light intensity. A control treatment was always kept in the dark. Mycelia were collected, dried on paper, frozen in liquid nitrogen, and stored at − 80 °C.

For the subcellular purification of proteins, a total of 10^7^ conidia were inoculated in 200 ml of Vogel’s liquid medium in 500-ml flasks, and incubated for 48 h at 30 °C under agitation in constant light, darkness, or darkness followed by exposure to light for 30 min. Mycelia were fixed by adding 540 μl of formaldehyde for 15 min, and then the reaction was stopped by adding glycine (125 mM). Formaldehyde was not used for subcellular purification of proteins from aerial hyphae. Mycelia were then collected by filtration, dried on paper, frozen in liquid nitrogen, and stored at − 80 °C.

For the cycloheximide and thiolutin treatments, 10^6^ conidia were inoculated on 25 ml Vogel’s liquid medium in 90-mm plates, which were incubated in complete darkness at 34 °C for 24 h to obtain a mycelial mat that was divided in disks of about 1.5 cm in diameter. Several disks were transferred to 200 ml of Vogel’s liquid medium in 500-ml flasks, and incubated for 24 h at 30 °C under agitation to allow further vegetative growth. Cultures were kept in constant light, darkness, or darkness followed by exposure to light for 30 min prior to addition of cycloheximide (0.1 mg/l) and/or thiolutin (50 μM). Mycelial disks were collected at time intervals after incubation with each chemical, dried on paper, frozen in liquid nitrogen, and stored at − 80 °C.

For the induction of conidiation, we modified the method of Bailey-Shrode and Ebbole [47]. A total of 10^7^ conidia were inoculated in 200 ml of Vogel’s liquid medium in 500-ml flasks, and incubated for 24 h at 30 °C under agitation. Vegetative mycelia were collected by filtration onto filter paper, covered by an additional layer of filter paper, and placed on top of 25 ml Vogel’s solid medium in 90-mm plates to induce conidiation. Cultures were incubated at 30 °C during 15 or 24 h when the aerial hyphae that developed and grew through the filter paper were collected, together with the supporting mycelial mat located between the two papers. Samples of vegetative mycelia from the initial liquid cultures were collected as vegetative controls. Cultures were either kept in the dark or under constant light. Samples were dried on paper, frozen in liquid nitrogen, and stored at − 80 °C.

Constant illumination was provided by a set of fluorescent lamps (containing 1 W/m^2^ of blue light). All the manipulations in the dark were performed under red light. Experiments were usually repeated at least three times and the specific number of repeats to obtain averages and standard errors in RNA or protein quantification experiments appears in the legend for each figure.

### Plasmid construction and gene replacement with *ve-1*^*FLAG*^

The generation of a *N. crassa* strain with VE-1 tagged by the FLAG epitope after replacement of the wild-type allele by the *ve-1*^*FLAG*^ allele was performed following the method described by [56] with minor modifications. Generation of knock-in tagging cassettes involved the generation of three DNA segments of about 1.2 kb in length: one segment corresponding to DNA upstream of the stop codon (5′-UTR of *ve-1*), a segment corresponding to the FLAG epitope and hygromycin resistance gene fusion (10xGly::3XFLAG::*hph*), and the final segment corresponding to DNA downstream of the stop codon (3′-UTR of *ve-1*). Each flank is obtained by PCR using primers (Additional file 3: Table S2) with a tail complementary to the tagging fragment obtained by digestion of plasmid DNA (plasmid p3xFLAG::hph::loxP; Genbank # FJ457009). The three fragments (two PCR products and a linear digested fragment) and the linearized plasmid pRS426 were fused to make the knock-in cassettes by recombination in an auxotrophic strain of *S. cerevisiae* [57]. The FLAG epitope was fused to VE-1 by a polyglycine adaptor, followed by the hygromycin resistance gene (*hph*) for the selection of transformed *N. crassa* strains. Transformants were backcrossed with the wild-type strain to isolate homokaryotic strains. Genetic crosses were performed to obtain strains that carried the *ve-1*^*FLAG*^ allele with selected mutations. All strains were verified by PCR and Western blots.

### Complementation of the Δ*ve-1* strain

The Δ*ve-1* strain was cotransformed with plasmid pNDN-OGG containing the nourseothricin resistance gene (*nat1*) under the control of the *A. nidulans trpC* promoter [58] and the wild-type allele of *ve-1* including 1625 bp upstream of the ATG and 1694 downstream of the stop codon (total length of 5049 bp) (PCR primers in Additional file 3: Table S2). Transformants were selected by growth in nourseothricin (50 μg/ml), and homokaryotic strains isolated after three cycles of vegetative growth, collection of conidia, and single-colony isolation. The presence of the wild-type *ve-1* gene in candidate strains was confirmed by PCR.

### RNA isolation and quantitative RT-PCR

Mycelia were disrupted by two 0.5 min pulses in a cell homogenizer (FastPrep-24, MP Biomedicals) with 1.5 g of zirconium beads (0.5 mm diameter) in 1.9 ml screw-cap tubes by using the RNeasy Plant Mini Kit (Qiagen) with the RLC buffer following the manufacturer procedure. The extracts in screw-cap tubes were clarified by centrifugation in a microcentrifuge (13,000 rpm) for 5 min prior to RNA purification. The RNA samples were treated with DNase I (USB) prior to use in RT-PCR experiments. The quality of the RNA was confirmed by inspecting the absorption spectra at 260 nm as recommended by the supplier of the RT-PCR kit and equipment. The primers employed for quantitative RT-PCR are detailed in Additional file 3: Table S2. Quantitative RT-PCR experiments were performed using one-step RT-PCR, in a LightCycler 480 II instrument (Roche) by using the One-Step SYBR® PrimeScript™ RT-PCR Kit (Takara Bio Inc.), 0.2 μM of each primer, and 50 ng of RNA in a 10 μl reaction. The reaction consisted of 5 min at 42 °C, followed by 10 s at 95 °C, and then 40 cycles of DNA amplification (5 s at 95 °C and 20 s at 60 °C). After each PCR, we performed melting curve analysis to show the specific amplification of single DNA segments and the absence of nonspecific amplified DNA. The fluorescent signal obtained for each gene was normalized to the corresponding fluorescent signal obtained with *tub-2* to correct for sampling errors. Expression data are the average of at least three independent experiments.

### Protein isolation and detection

Proteins were extracted from mycelia by previously described methods [59] using a modified lysis buffer (50 mM HEPES pH 7.4, 137 mM NaCl, 10% glycerol, 5 mM EDTA, 29.3 μM phenylmethyl-sulphonylfluoride (PMSF), 6.3 μM leupeptin, 4.4 μM pepstatin A, and phosphatases inhibitors (PhosStop, Roche) when indicated. Total proteins were subjected to SDS-PAGE on 7.5% (30:0.2) acrylamide-bisacrylamide gels and transferred to nitrocellulose membranes. Equal loading was confirmed by staining the hybridization membrane with Pounceau S solution. Membranes were hybridized with polyclonal antibodies against WC-1, monoclonal against FLAG (Sigma), polyclonal against histone 3 (H3) (Abcam), monoclonal against tubulin (Santa Cruz), or monoclonal against NOP1p (EnCor Biotechnology). Horseradish peroxidase-conjugated anti-rabbit IgG (Bio-Rad) was used as secondary antibody. Antibody binding was observed by chemiluminescence (GE Healthcare).

Nuclear fraction purifications were performed as developed by Baum and Giles [60] with minor modifications by [14, 61]. Frozen mycelia (4–5 g) were pulverized to a fine powder in liquid nitrogen. Eight milliliters of buffer A [1 M sorbitol, 7% (w/v) Ficoll, 20% (v/v) glycerol, 5 mM magnesium acetate, 5 mM EGTA, 3 mM calcium chloride, 50 mM Tris–HCl pH 7.5] was added to the powder. Once homogenized, the mixture was filtered through gauze and two volumes of buffer B [10% (v/v) glycerol, 5 mM magnesium acetate, 5 mM EGTA, 25 mM Tris–HCl pH 7.5] were added to the filtrate. The mix of filtrate and buffer B was slowly added to 10.4 ml buffer C (1:1.7 Buffers A:B) as to prevent the liquid phases from mixing. This step was performed on 25 × 89 mm centrifuge tubes (Beckman). Centrifugation was performed at 3000 × *g*, 4 °C. A 1-ml sample was collected from the supernatant and labelled total cell extract. The remainder was added to 5 ml of 1 M sucrose gradient [1 M sucrose, 10% (v/v) glycerol, 5 mM magnesium acetate, 1 mM DTT, 25 mM Tris–HCl, pH 7.5] slowly as to prevent mixing of liquid phases. Centrifugation was performed for 30 min at 9400 × *g*, 4 °C. The resulting supernatant was collected and labelled cytoplasmic fraction. The pellet was suspended in 500 μl buffer D [25% (v/v) glycerol, 5 mM magnesium acetate, 3 mM DTT, 0.1 mM EDTA, 25 mM Tris–HCl pH 7.5] and labelled as nuclear fraction. Samples for total cell extract, cytoplasmic fraction, and nuclear fraction were quantified.

### Co-immunoprecipitation experiments

Co-immunoprecipitations of VE-1 for detection of possible interactions with WC-1 were performed as follows. The strain with the v*e-1*^*FLAG*^ allele was grown for 48 h as described for the cellular fractionation experiments. Mycelia were fixed with formaldehyde and collected as described. Mycelial pads (8–10 g wet weight) were harvested by filtration, frozen in liquid nitrogen, and ground to fine powders, which were mixed in a 50-ml tube with 20 ml of IPB buffer [50 mM Tris–HCl pH 7.5, 150 mM NaCl, 1.5 mM MgCl2, 0.1% (v/v) NP-40] with protease inhibitors (Roche complete protease inhibitor cocktail) and dissolved by vortexing on ice. After mixing, crude extracts were centrifuged at 14,300 × *g*, at 4 °C for 20 min. Supernatants were collected and transferred to ultracentrifuge tubes. Centrifugation was performed at 169,000 × *g* at 4 °C for 45 min. Then, a 1-ml fraction of each supernatant was transferred to a new tube, labeled “INPUT,” frozen in liquid nitrogen, and stored at − 80 °C until use. The remaining supernatant was collected in 15-ml screw-cap tubes and kept at 4 °C. During the 45-min centrifugation, 300 μl of M2-FLAG agarose beads (Sigma) was equilibrated by washing them three times with 1 ml of IPB buffer followed by centrifugation at 10000 × *g* at 4 °C. After equilibration, the beads were added to the supernatant in 15-ml tubes, and the mix was incubated for 3 h at 4 °C in a rotating mixer. Beads were then precipitated by centrifuging at 1500 × *g* for 10 min at 4 °C. A 1-ml sample of each supernatant was transferred to a new tube, labeled “flowthrough” (FT), frozen in liquid nitrogen, and stored at − 80 °C. The beads were washed twice with 10 ml of IPB buffer by gentle rotation for 4 min at 4 °C and finally suspended with 500 μl of IPB buffer and transferred to new 2-ml tubes. Beads were washed twice with 1 ml IPB buffer by centrifugation at 1000 × *g* at 4 °C. At the end, 120 μl of NuPAGE buffer (Invitrogen) was added to the beads, vortexed, and incubated for 10 min at 72 °C with moderate shaking. The supernatant was collected by centrifuging at 1000 × *g* at 4 °C and transferred to new tubes labeled “IP.” Samples were treated with DTT (50 mM) and incubated again for 10 min at 72 °C. Protein concentrations from the INPUT samples were measured in a Nanodrop instrument (Absorbance set to 280 nm). INPUT, FT, and IP samples were used for western blot as described previously.

### Carotenoid analysis

Carotenoid analysis was performed as described earlier [62]. Mycelia were collected from the liquid medium by filtration and conidia were collected from conidiating cultures, and cleaned with sterile water followed by centrifugation for conidia collection. Mycelia or conidia were immediately frozen in liquid nitrogen and freeze-dried for 24 h. Carotenoids were extracted with acetone in a FastPrep-24 device (MP Biomedicals), dried in a Concentrator Plus equipment (Eppendorf), and dissolved in 0.1–1 ml of n-hexane. Spectrophotometrical measurements were recorded from 350 to 650 nm in a Shimadzu UV spectrophotometer 1800. Total amounts of carotenoids were estimated according to the maximal absorbance at ca. 480 nm of the sample and an average maximal E of (1 mg l^−1^, 1 cm) of 200. Final values were corrected to the dry weight and dilution of the sample.

### Conidiation

For the quantification of conidiation, we prepared 50-ml tubes with 7.5 ml of Vogel’s solid medium. Each tube was inoculated with 10^6^ conidia and incubated at 30 °C in dark or constant light. Conidia were isolated after addition of 5 ml of water and gentle shaking to each tube. Each conidial suspension was collected, centrifuged, and resuspended in sterile water three times prior to quantification in a hemocytometer.

## Supplementary Information

**Additional file 1:** **Figure S1**. Carotenoid and hyphal growth phenotypes of the wild type, the *ve-1* mutant and the Δ*ve-1* mutant complemented with a wild-type copy of *ve-1* (Δ*ve-1*C). **Figure S2.** VE-1 is required for the accumulation of carotenoids and accumulates in vegetative hyphae. **Figure S3.** Conidiation in the wild type and Δ*ve-1* mutant. **Figure S4.** Conidiation in *N. crassa*. **Figure S5.** Stability and degradation of the components of the velvet complex and WC-1. **Figure S6.** Mutations in the protein degradation pathway do not modify the light-dependent phosphorylation of WC-1 or the subcellular localization of VE-1.

**Additional file 2:** **Table S1.** *N. crassa* strains used in this study.

**Additional file 3:** **Table S2.** Oligonucleotides used in this study.

**Additional file 4:** Individual values for figures where the number of independent replicates is less than 6.

**Additional file 5:** Uncropped images or original hybridization films for western blots.

## Data Availability

The data supporting the findings of this study are presented within the manuscript and the additional supporting files of this article. *N. crassa* mutants are available from the Fungal Genetics Stock Center at the University of Missouri, Kansas City (www.fgsc.net), or from the authors on reasonable request.

## Abbreviations

CRL: Cullin-RING ubiquitin ligase
CSN: COP9 signalosome
JAMM: JAB1/MPN/Mov34
MAP: Mitogen-activated protein
PCI: Proteasome, COP9 signalosome, eukaryotic initiation factor 3
PEST: Protein segment rich in proline (P), glutamic acid (E), serine (S), and threonine (T)
SCF: SKP Cullin F-box
UPS: Ubiquitin–proteasome system
WCC: White Collar Complex
