# Supplementary material for: Light regulates the degradation of the regulatory protein VE-1 in the fungus Neurospora crassa
Source: BMC Biol. 2022 Jun 27;20:149. doi: 10.1186/s12915-022-01351-x (PMC9238092; doi:10.1186/s12915-022-01351-x)
Supplement: Supplementary file 1 — Additional file 1: Figure S1. Carotenoid and hyphal growth phenotypes of the wild type, the ve-1 mutant and the Δve-1 mutant complemented with a wild-type copy of ve-1 (Δve-1C). Figure S2. VE-1 is required for the accumulation of carotenoids and accumulates in vegetative hyphae. Figure S3. Conidiation in the wild type and Δve-1 mutant. Figure S4. Conidiation in N. crassa. Figure S5. Stability and degradation of the components of the velvet complex and WC-1. Figure S6. Mutations in the protein degradation pathway do not modify the light-dependent phosphorylation of WC-1 or the subcellular localization of VE-1. [file 12915_2022_1351_MOESM1_ESM.pdf]

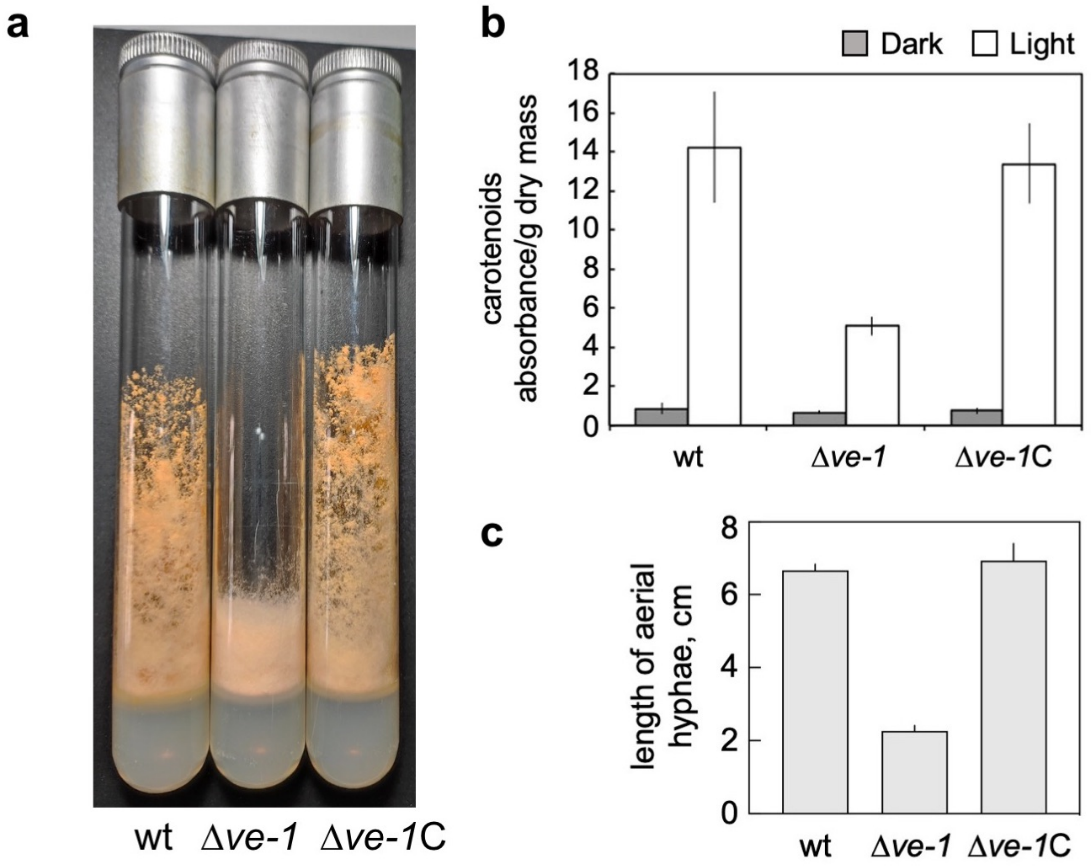


**Fig. S1.** Carotenoid and hyphal growth phenotypes of the wild type, the *ve-1* mutant and the Δ*ve-1* mutant complemented with a wild-type copy of *ve-1* (Δ*ve-1*C). a, Growth phenotype of the strains after four days at 30ºC in light. b, Accumulation of carotenoids in mycelia of the strains grown for two days in the dark at 22 ºC and illuminated for 30 min (1 W/m$^2$ of blue light) prior to incubation for 24 h in the dark at 8 ºC. The amount of carotenoids was estimated as the absorbance at 475 nm and corrected to the dry mass of each sample. The plot shows the average and standard error for 2-3 experiments. c, Length of aerial hyphae in each strain. Average and standard error of the mean in three independent experiments after four days of growth at 30ºC in light.

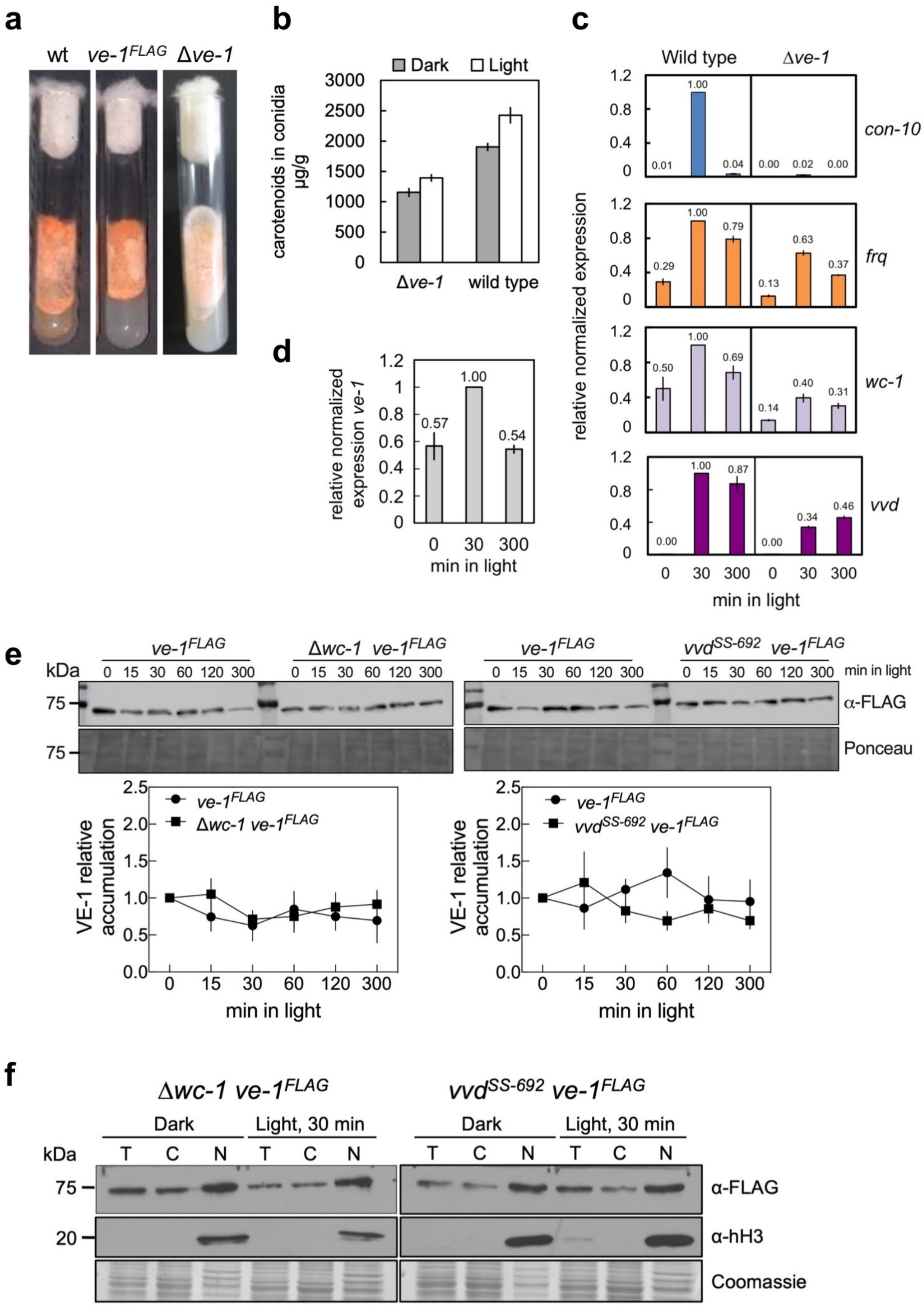


**Fig. S2.** VE-1 is required for the accumulation of carotenoids and accumulates in vegetative hyphae. a, Growth phenotype of the wild-type, the *ve-1*$^{FLAG}$, and the Δ*ve-1* mutant strain. b, Accumulation of carotenoids in conidia of the Δ*ve-1* mutant and wild-type strain. Mycelia of each strain were

grown in dark or light at 30ºC for three days before collecting conidia. The plot shows the average and standard error of six independent experiments. c, Light-dependent transcription and photoadaptation in the wild type and Δ*ve-1* mutant. Wild-type and Δ*ve-1* mutant strains were grown for two days at 22ºC in the dark and then exposed to light during the times indicated prior to RNA purification and quantification by RT-PCR (time 0 represent mRNAs from mycelia kept in the dark as controls). The plots show the average and standard error of the mean of the relative mRNA accumulation in 3-6 independent experiments. The results from each PCR for each gene were normalized to the corresponding PCR for *tub-2* to correct for sampling errors. Then, the results were normalized to those obtained with the wild type after exposure to 30 min of light. d, Light-dependent transcription and photoadaptation of gene *ve-1*. The wild-type strain was grown for two days at 22ºC in the dark and then exposed to light during the times indicated prior to RNA purification and quantification by RT-PCR (time 0 represent mRNAs from mycelia kept in the dark as controls). The plots show the average and standard error of the mean of the relative mRNA accumulation in three independent experiments. The results from each PCR for each gene were normalized to the corresponding PCR for *tub-2* to correct for sampling errors. Then, the results were normalized to those obtained after exposure to 30 min of light. e, VE-1 accumulates in vegetative mycelia of the wild type and photoreceptor mutants. Proteins were isolated from mycelia of the $ve\text{-}1^{FLAG}$, Δ*wc-1* $ve\text{-}1^{FLAG}$, and $vvd^{S\text{-}992}$ $ve\text{-}1^{FLAG}$ strains grown for two days at 22ºC in the dark and then exposed to light during the times indicated. Proteins were separated by SDS-PAGE, and hybridized with an antibody specific for FLAG. 30 µg of proteins were loaded per lane. As loading control we used a Ponceau staining of each protein sample. Each hybridization was quantified using as reference the amount of VE-1 detected at time 0 (mycelia kept in the dark). The plot shows the average and standard error of three independent experiments. f, Subcellular localization of VE-1 in photoreceptor mutants. Mycelial samples of the Δ*wc-1* $ve\text{-}1^{FLAG}$, and $vvd^{S\text{-}992}$ $ve\text{-}1^{FLAG}$ strains were grown for two days at 30ºC in the dark, or grown in the dark and exposed to light during 30 min. Total protein samples (T), or samples enriched in

cytoplasmic (C) or nuclear (N) proteins were separated by SDS-PAGE, and hybridized with antibodies specific for FLAG or histone H3. 70 µg of proteins were loaded per lane. As loading control we used a Coomassie staining of each protein sample.

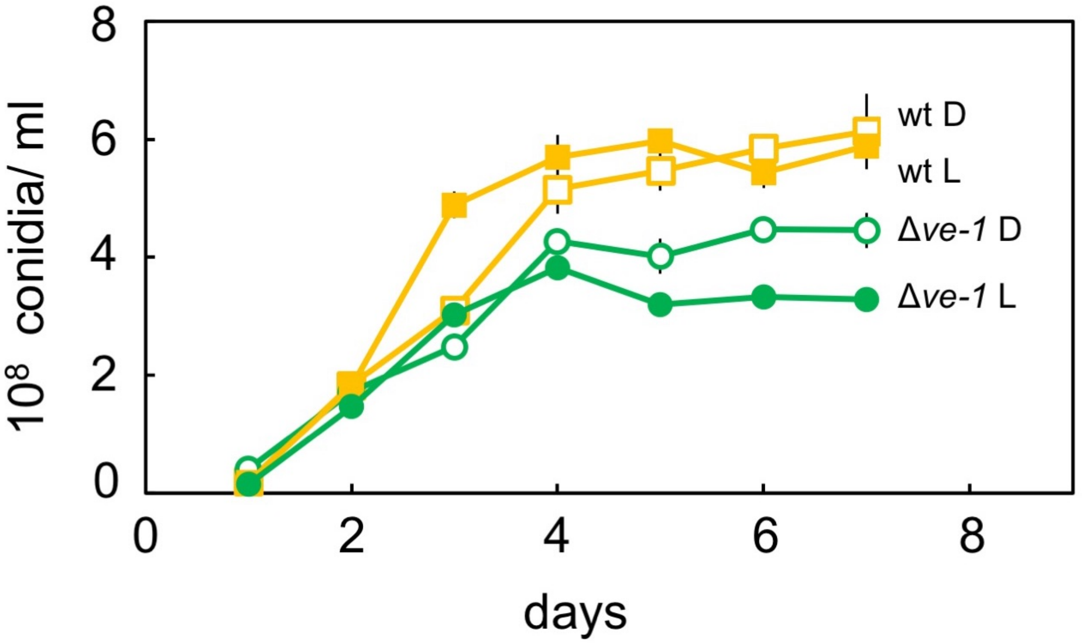


**Fig. S3.** Conidiation in the wild type and Δ*ve-1* mutant. Mycelia of each strain were grown in dark (D) or light (L) at 30ºC and conidia collected at the indicated times. The plot shows the average and standard error of three independent experiments.

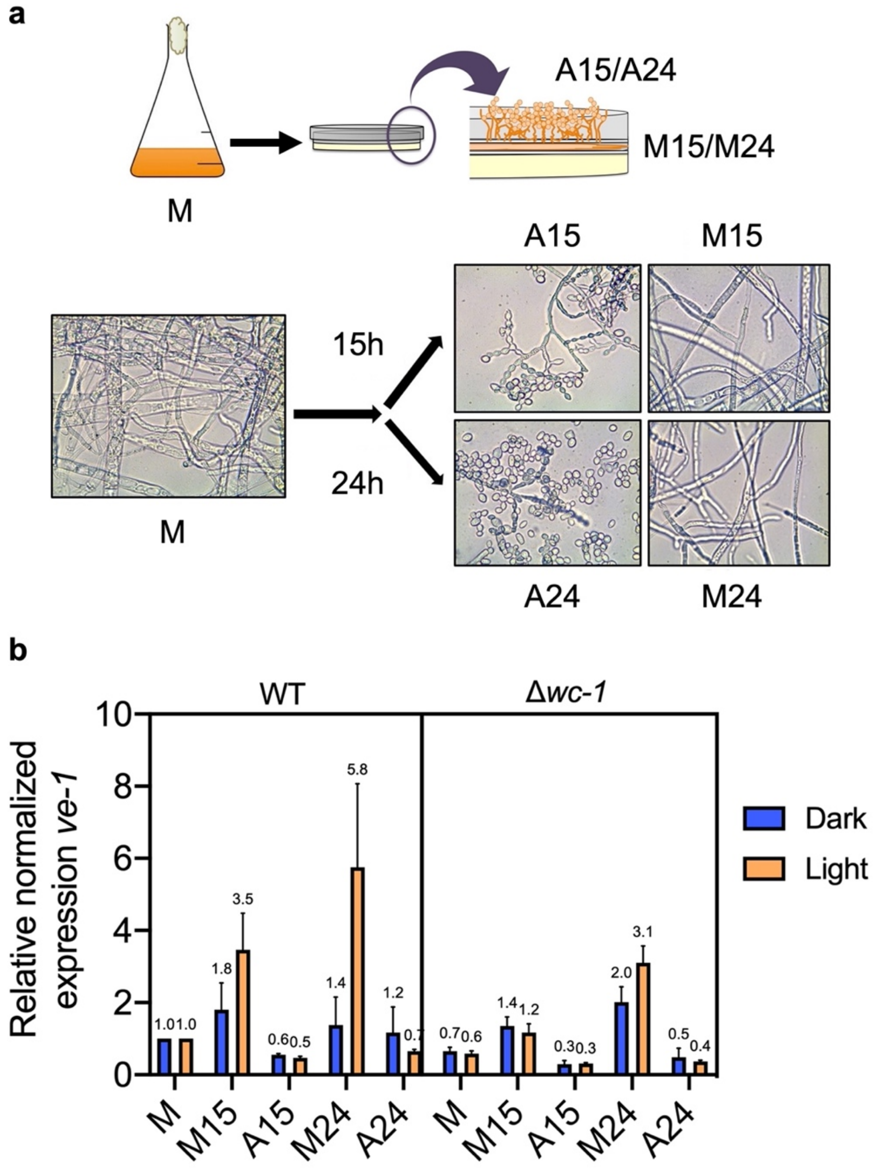


**Fig. S4.** Conidiation in *N. crassa*. a, Experimental design to characterize conidiation. Upper part. Mycelia was grown vegetatively in liquid media during 24 h (M). We then transferred the hyphae from liquid media to the air-exposed surface of a petri dish with solid minimal agar to induce conidiation, and covered the vegetative mycelia with filter paper. After 15 h or 24 h of the induction of conidiation we collected the supportive vegetative mycelia (M15 and M24) and the aerial hyphae and conidia (A15 and A24) that grew through the filter paper. Bottom part. Images from each sample were taken with a microscope with 20x magnification. b, The mRNA of *ve-1* accumulates during conidiation. RNA samples from cultures kept in the dark or light were obtained from wild-type mycelia growing vegetatively in liquid media (M), or as

supporting vegetative mycelia after 15 h (M15) or 24 h (M24) of transfer from liquid media to the surface of an agar plate with minimal media to induce conidiation. Conidiating aerial hyphae were collected after 15 h (A15) or 24 h (A24) of the induction of conidiation. Total RNA were purified and quantified by RT-PCR. The plots show the average and standard error of the mean of the relative mRNA accumulation in three independent experiments. The results from each PCR for each gene were normalized to the corresponding PCR for *tub-2* to correct for sampling errors. Then, the results were normalized to those obtained with the wild type in vegetative mycelia (M).

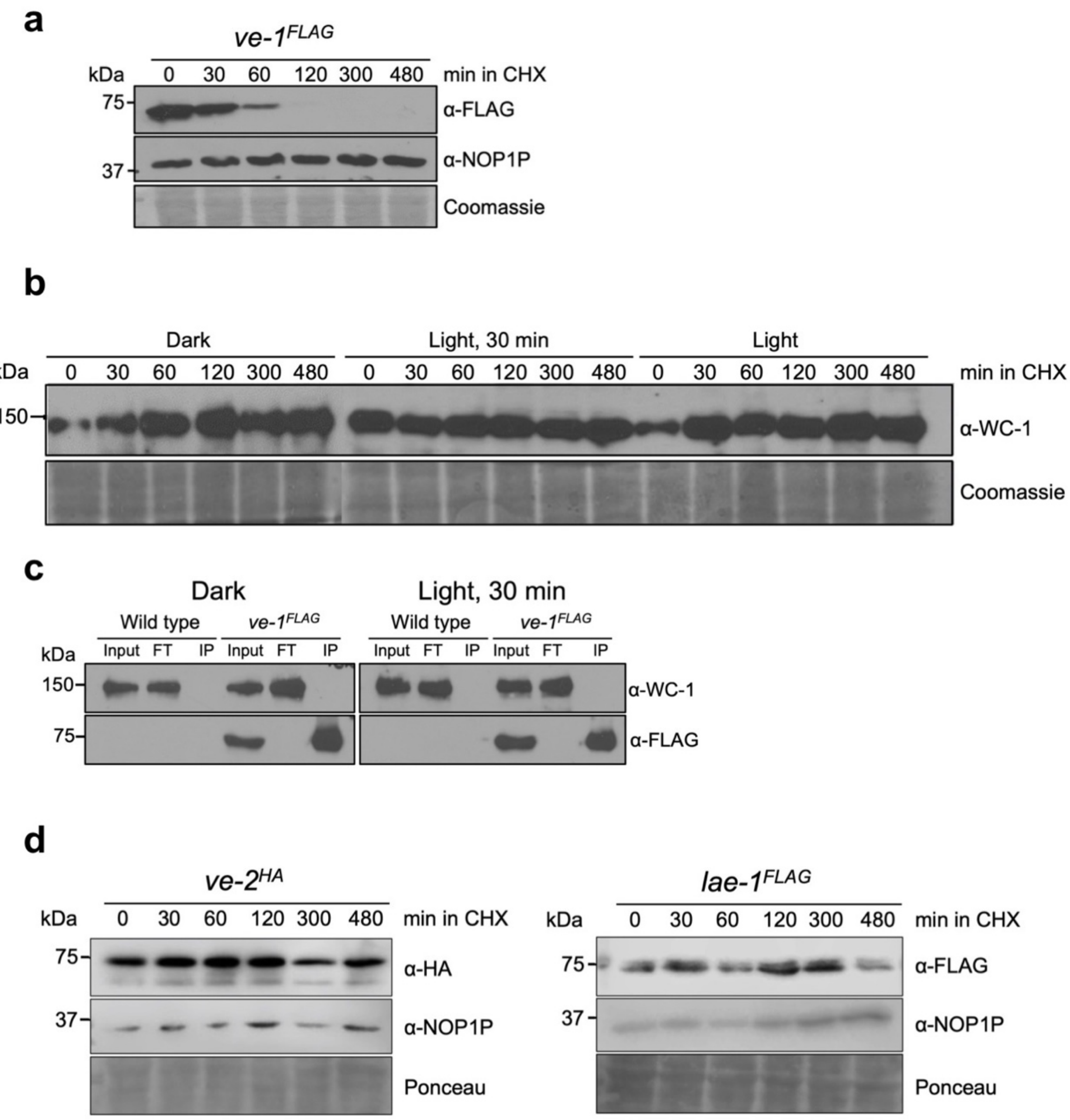


**Fig. S5.** Stability and degradation of the components of the velvet complex and WC-1. a, Degradation of VE-1. Cultures of vegetative mycelia of the *ve-1*$^{FLAG}$ strain were grown at 30ºC in liquid media for 24h in the dark and exposed to 30 min of light, then cycloheximide was added to the cultures, and samples were removed at different times. Proteins were separated by SDS-PAGE, and hybridized with an antibody specific for FLAG or NOP1P. 70 µg of proteins were loaded per lane. As loading control we used a Coomassie staining of each protein sample. b, WC-1 is a stable protein. Cultures of vegetative mycelia of the *ve-1*$^{FLAG}$ strain were grown at 30ºC in liquid media for 24h in the dark, light or exposed to 30 min of light, then cycloheximide was added to the cultures, and samples were removed at different times. Proteins were separated by SDS-PAGE, and hybridized with an antibody specific for

WC-1. 70 µg of proteins were loaded per lane. As loading control we used a Coomassie staining of each protein sample. c, VE-1 and WC-1 do not interact. Cultures of vegetative mycelia of the wild-type and *ve-1*$^{FLAG}$ strains were grown at 30ºC in liquid media for 48 h in the dark or exposed to 30 min of light. VE-1 was immunopurified using an antibody against FLAG, and the column samples were separated by SDS-PAGE, and hybridized with an antibody specific for FLAG or WC-1. 70 µg of proteins were loaded per lane. We used the input, flow-through (FT), and immunoprecipitated samples (IP). d, VE-2 and LAE-1 are stable proteins. Cultures of vegetative mycelia of the *ve-2*$^{HA}$ or the *lae-1*$^{FLAG}$ strains were grown at 30ºC in liquid media for 24h in light, then cycloheximide was added to the cultures, and samples were removed at different times. Proteins were separated by SDS-PAGE, and hybridized with an antibody specific for FLAG, HA, or NOP1P. 30 µg of proteins were loaded per lane. As loading control we used Ponceau staining of each protein sample.

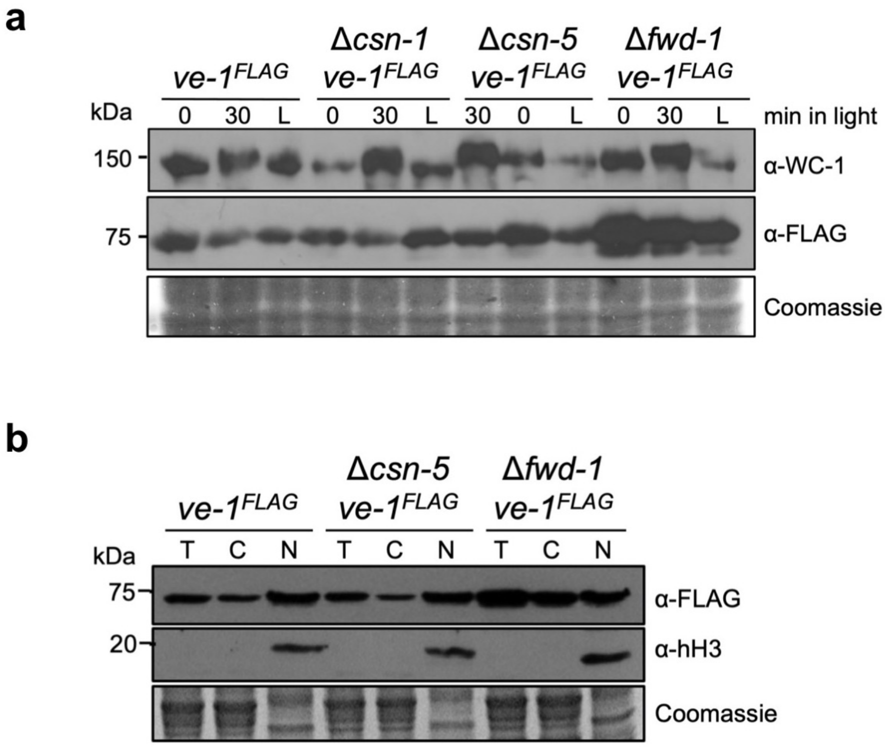


**Fig. S6.** Mutations in the protein degradation pathway do not modify the light-dependent phosphorylation of WC-1 or the subcellular localization of VE-1. a, Light-dependent phosphorylation of WC-1 in mutants of the protein degradation pathway. Mycelial samples of the *ve-1*$^{FLAG}$, Δ*csn-1 ve-1*$^{FLAG}$, Δ*csn-5 ve-1*$^{FLAG}$ and Δ*fwd-1 ve-1*$^{FLAG}$ strains were isolated from cultures kept in the dark, light or exposed for 30 min of light. Total protein extracts were separated by SDS-PAGE, and hybridized with an antibody specific for FLAG or WC-1. 200 µg of proteins were loaded per lane. Additional bands in the WC-1 hybridization are due to the transient light-dependent WC-1 phosphorylation. As loading control we used a Coomassie staining of each protein sample. b, Subcellular localization of VE-1 in mutants of the protein degradation pathway. Mycelial samples of the *ve-1*$^{FLAG}$, Δ*csn-5 ve-1*$^{FLAG}$ and Δ*fwd-1 ve-1*$^{FLAG}$ strains were grown for two days at 30ºC in light. Total protein samples (T), or samples enriched in cytoplasmic (C) or nuclear (N) proteins were separated by SDS-PAGE, and hybridized with antibodies specific for FLAG or histone H3. 70 µg of proteins were loaded per lane. As loading control we used a Coomassie staining of each protein sample.
