# Supplementary material for: Light regulates the degradation of the regulatory protein VE-1 in the fungus Neurospora crassa
Source: BMC Biol. 2022 Jun 27;20:149. doi: 10.1186/s12915-022-01351-x (PMC9238092; doi:10.1186/s12915-022-01351-x)
Supplement: Supplementary file 2 — Additional file 2: Table S1. N. crassa strains used in this study. [file 12915_2022_1351_MOESM2_ESM.pdf]

Table S1. *N. crassa* strains used in this study

Strain	Genotype	Source
FGSC#2489 (wild type)	74-OR23-1VA	FGSC
FGSC#11401	Δ*ve-1 hyg*^{R}	FGSC
VE-1FLAG	*ve-1*^{FLAG} *hyg*^{R}	This study
VE-1C	Δ*ve-1 ve-1 hyg*^{R} *nat*^{R}	This study
VE-2HA	*ve-2*^{HA} *hyg*^{R}	Bayram el al.
LAE-1FLAG	*lae-1*^{FLAG} *hyg*^{R}	Bayram et al.
ve-1 wc-1	Δ*ve-1* Δ*wc-1 hyg*^{R}	This study
wc-1 VE-1FLAG	Δ*wc-1 ve-1*^{FLAG} *hyg*^{R}	This study
csn-1 VE-1FLAG	Δ*csn-1 ve-1*^{FLAG} *hyg*^{R}	This study
csn-5 VE-1FLAG	Δ*csn-5 ve-1*^{FLAG} *hyg*^{R}	This study
fwd-1 VE-1FLAG	Δ*fwd-1 ve-1*^{FLAG} *hyg*^{R}	This study
vvd VE-1FLAG	*vvd*$^{SS\text{-}692}$ *ve-1*^{FLAG} *hyg*^{R}	This study
