# Supplementary material for: Light regulates the degradation of the regulatory protein VE-1 in the fungus Neurospora crassa
Source: BMC Biol. 2022 Jun 27;20:149. doi: 10.1186/s12915-022-01351-x (PMC9238092; doi:10.1186/s12915-022-01351-x)
Supplement: Supplementary file 3 — Additional file 3: Table S2. Oligonucleotides used in this study. [file 12915_2022_1351_MOESM3_ESM.pdf]

Table S2. Oligonucleotides used in this study

Designation	Sequence direction 5→3	Features
con10-45F	CAGCCACAGCGGAGGC	RT-PCR
con10-104R	TTGGAGCAATTTCGCGC	RT-PCR
ve1-939F	CGGAGGCCATCTGGGTTT	RT-PCR
ve1-998R	GGTGGTTGAGCGGGATACTG	RT-PCR
al1-226F	TCCAATGTTTCCCCAACTACAAC	RT-PCR
al1-328R	CGGTGGTGGGCGAGAA	RT-PCR
al2-F	CGCTATCGCCTACCCCATT	RT-PCR
al3-165F	CATCTCTTCCGCCGGTCTAG	RT-PCR
al3-227R	ACCGAGGCCTTGCGTTTAC	RT-PCR
cao2-F	TCAAGGGACTGAGAGAGCCG	RT-PCR
cao2-R	CGTTGACGTTGTTGTGCCAC	RT-PCR
tub-1073F	CCCGCGGTCTCAAGATGT	RT-PCR
tub-1139R	CGCTTGAAGAGCTCCTGGAT	RT-PCR
ve1 flank 5'F	GTA ACG CCA GGG TTT TCC CAG TCA CGA CGG GGT CGT CTG CAG ACT CCT TCG	ve1 FORWARD 5 UTR
ve1 flank 5'R	CCTC CGC CTC CGC CTC CGC CGC CTC CGC CAT ACC CGC CAA TAT CTG CCT GC	ve1 REVERSE 5 UTR
ve1 flank 3'F	TGC TAT ACG AAG TTA TGG ATC CGA GCT CGA CGG TTA TGT TCT TTG TGG GAT TTC TT	ve1 FORWARD 3 UTR
ve1 flank 3'R	GCG GAT AAC AAT TTC ACA CAG GAA ACA GCC AGG CCA CTT CAC TAA CCG GTC C	ve1 REVERSE 3 UTR
5Fve-1C	CTGCCCTGTGTATCCCATCC	Amplification of *ve-1* for complementation
3Rve-1C	GGACTCTGTTACCGCTCAAGCC	Amplification of *ve-1* for complementation
