# Supplementary material for: Light regulates the degradation of the regulatory protein VE-1 in the fungus Neurospora crassa
Source: BMC Biol. 2022 Jun 27;20:149. doi: 10.1186/s12915-022-01351-x (PMC9238092; doi:10.1186/s12915-022-01351-x)
Supplement: Supplementary file 5 — Additional file 5: Uncropped images or original hybridization films for western blots. [file 12915_2022_1351_MOESM5_ESM.pdf]

Uncropped images or original hybridization films for western blots

Fig. 1C

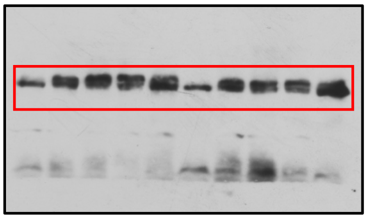

α-WC-1

Fig. 1D

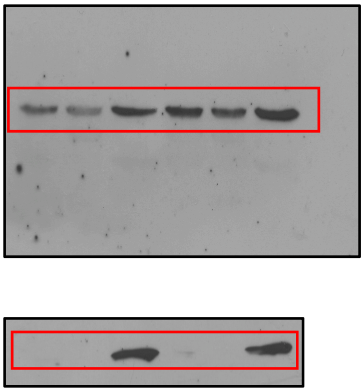

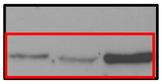

α-FLAG

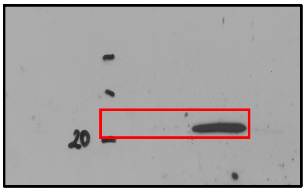

α-hH3

Fig. 2C

ve-1^FLAG

Δwc-1 ve-1^FLAG

Dark

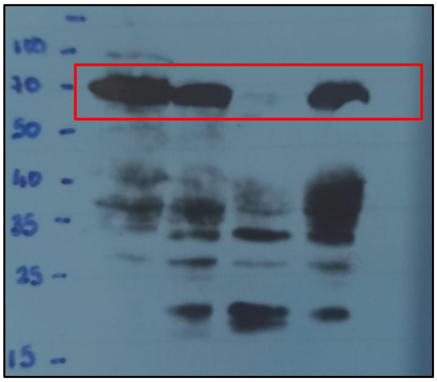

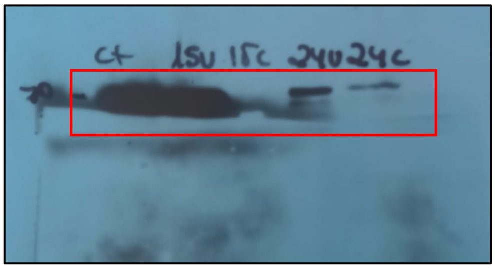

α-FLAG

Light

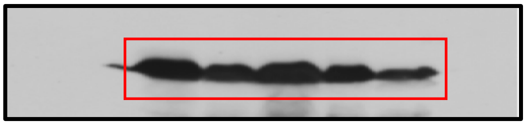

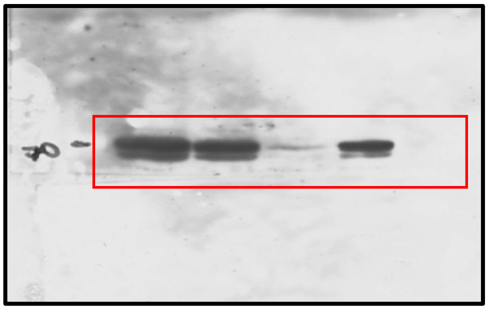

Fig. 2D

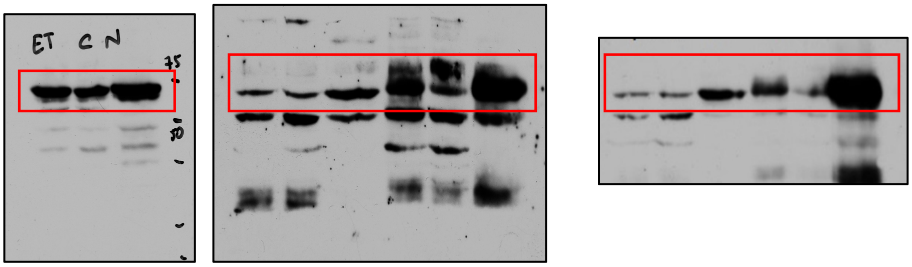

α-FLAG

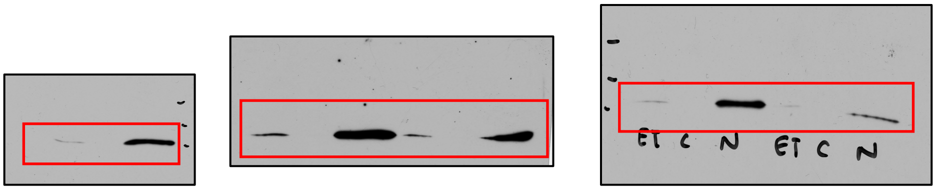

α-hH3

Fig. 3A

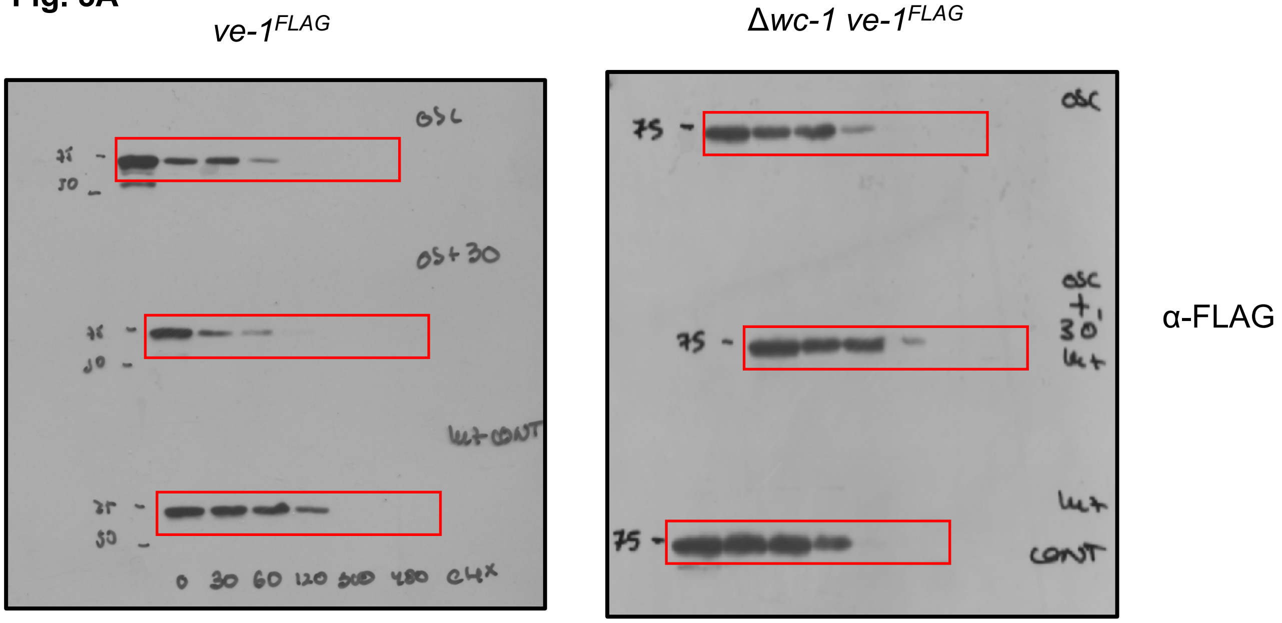

Fig. 3B

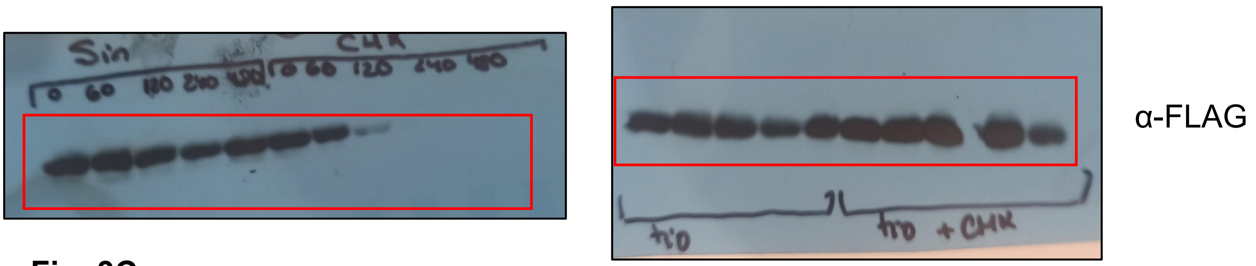

Fig. 3C

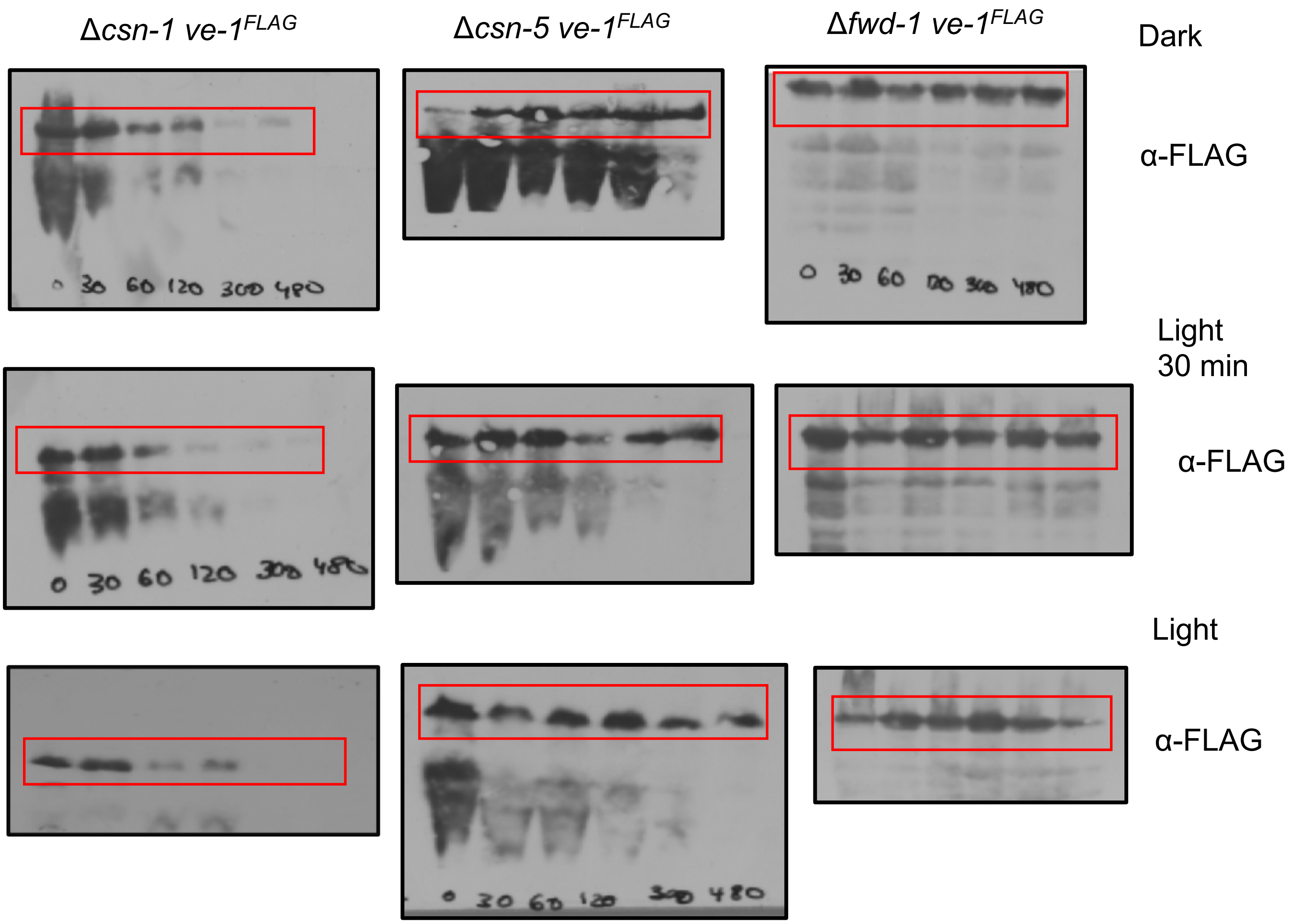

Fig. 4A

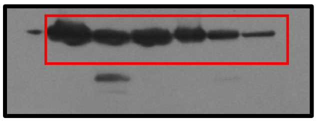

Fig. 4B

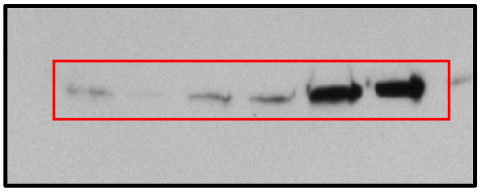

α-FLAG

Fig. 4C

Dark to light transfer

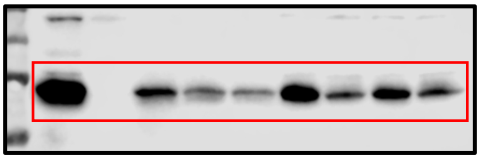

Light to dark transfer

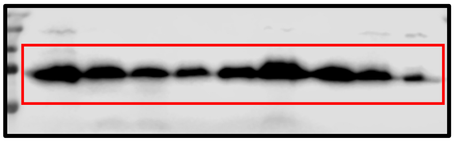

α-FLAG

Fig. S2E

ve-1^{FLAG} Δ*wc-1 ve-1*^{FLAG}

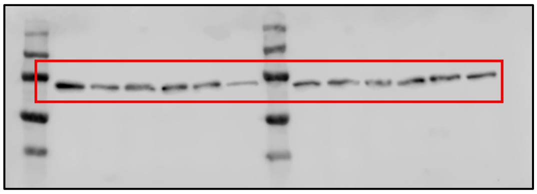

ve-1^{FLAG} *vvd*$^{SS\text{-}692}$ *ve-1*^{FLAG}

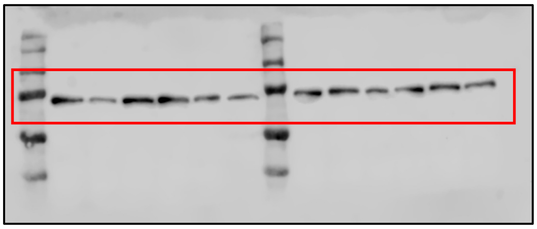

α-FLAG

Fig. S2F

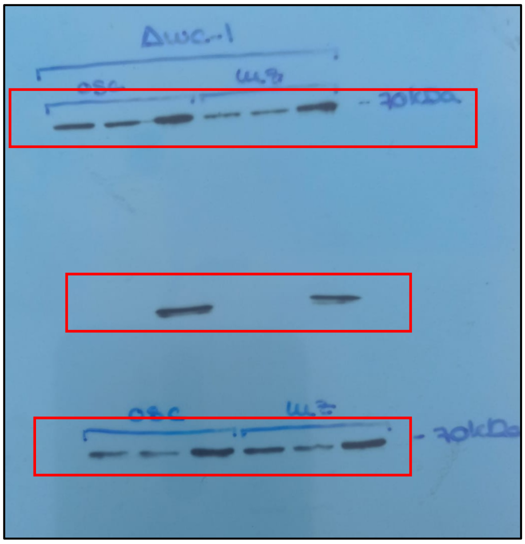

Δ*wc-1 ve-1*^{FLAG} α-FLAG

Δ*wc-1 ve-1*^{FLAG} α-hH3

vvd$^{SS\text{-}692}$ *ve-1*^{FLAG} α-FLAG

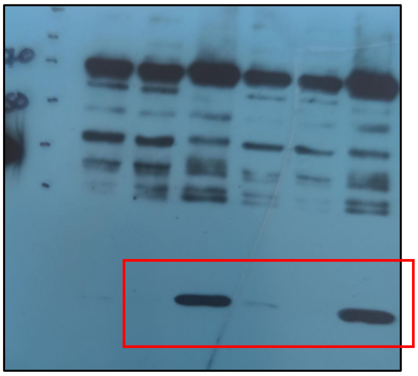

vvd$^{SS\text{-}692}$ *ve-1*^{FLAG} α-hH3

Fig. S5A

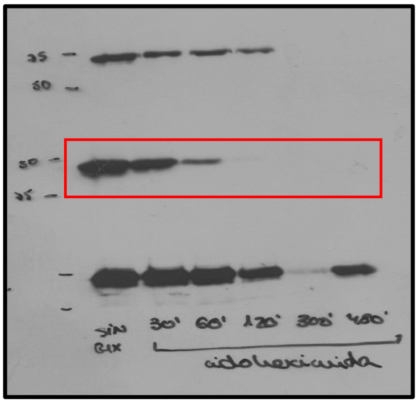

α-FLAG

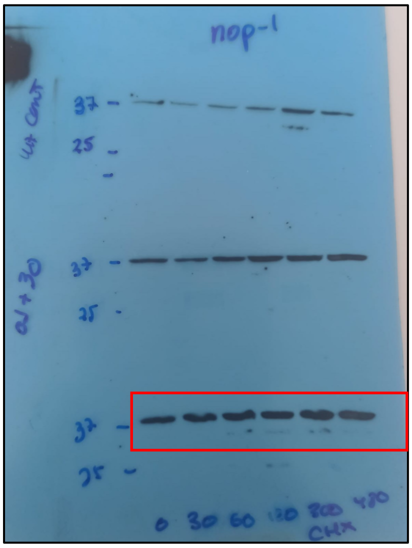

α-NOP1P

Fig. S5B

Dark

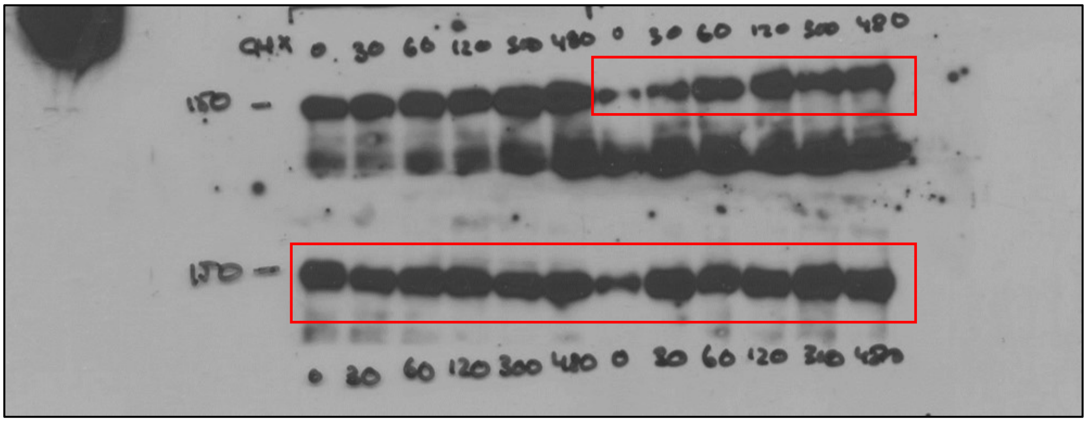

α-WC-1

Light, 30 min

Light

Fig. S5C

α-WC-1

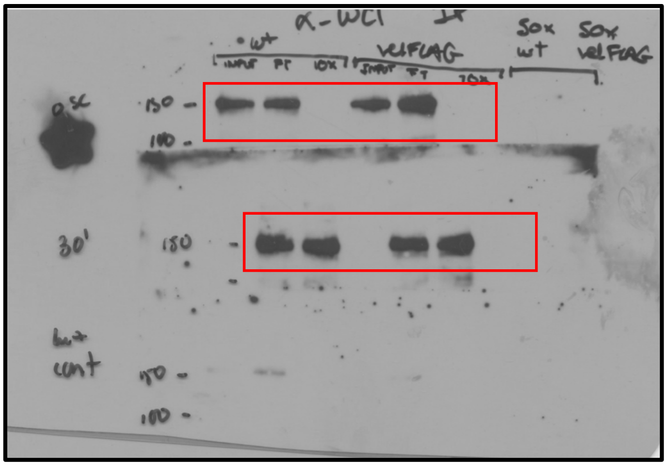

Dark

Light, 30 min

α-FLAG

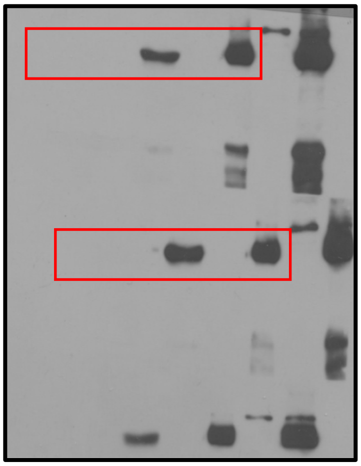

Fig. S5D

α-FLAG α-NOP1P

ve-2^{FLAG}

lae-1^{FLAG}

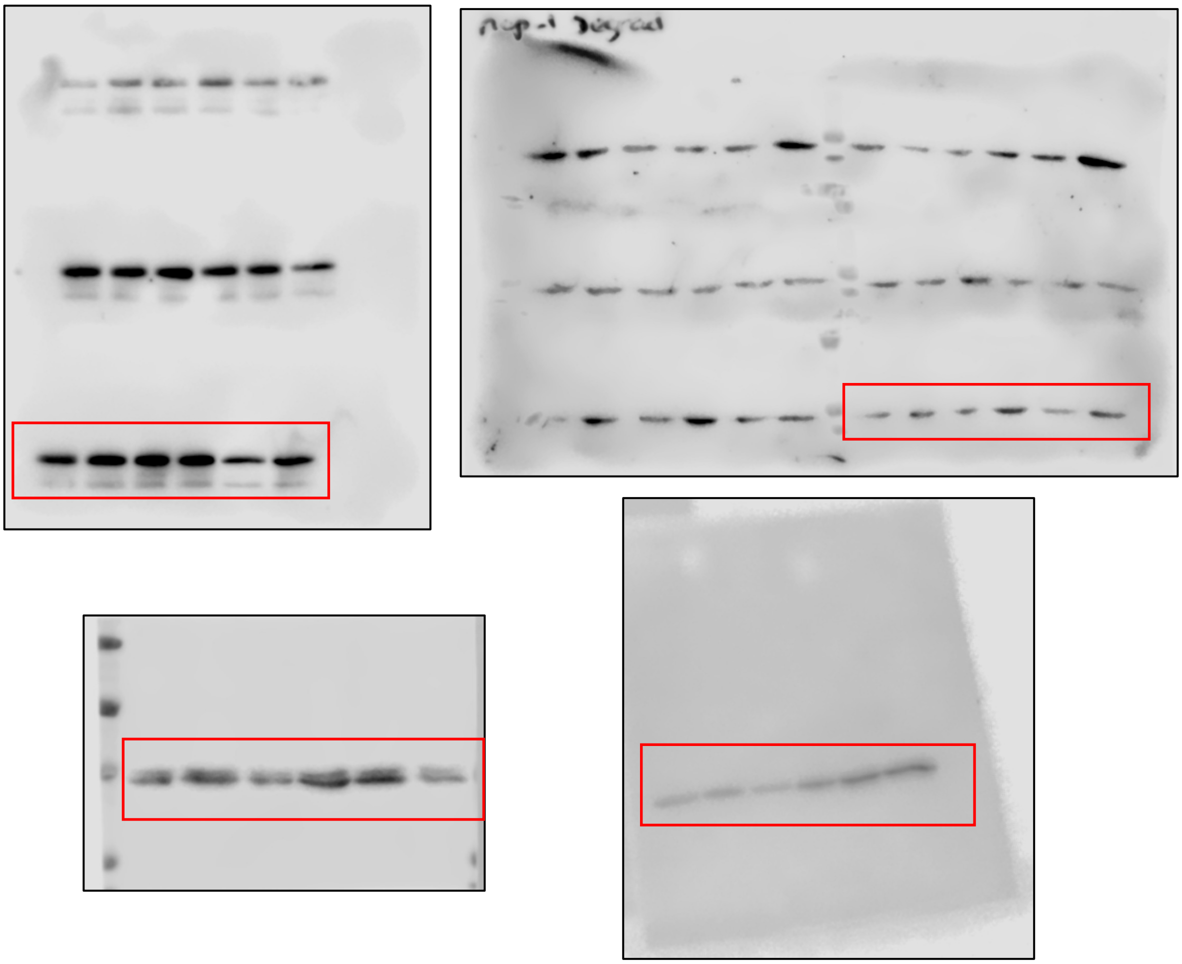

Fig. S6A

α-WC-1 α-FLAG

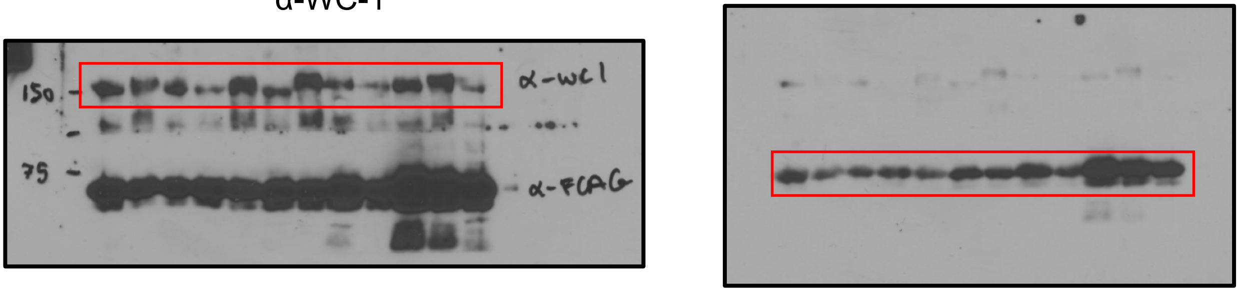

Fig. S6B

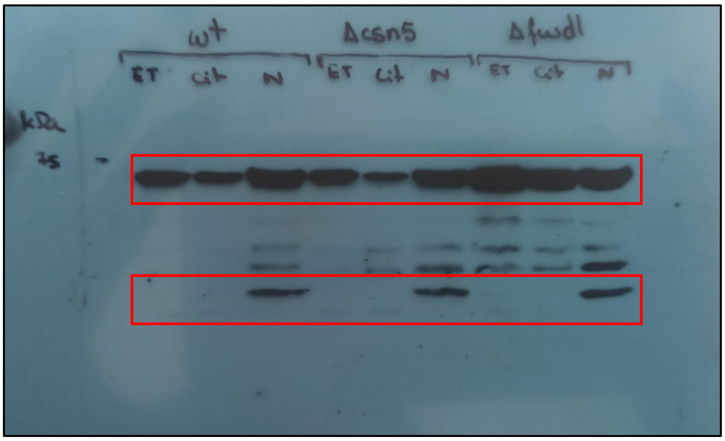

α-FLAG

α-hH3
